# Spatial transcriptomics reveals strong association between *SFRP4* and extracellular matrix remodeling in prostate cancer

**DOI:** 10.1038/s42003-024-07161-x

**Published:** 2024-11-08

**Authors:** Maria K. Andersen, Sebastian Krossa, Elise Midtbust, Christine A. Pedersen, Maximilian Wess, Therese S. Høiem, Trond Viset, Øystein Størkersen, Ingunn Nervik, Elise Sandsmark, Helena Bertilsson, Guro F. Giskeødegård, Morten B. Rye, May-Britt Tessem

**Affiliations:** 1 Department of Circulation and Medical Imaging, NTNU - Norwegian University of Science and Technology, Trondheim, Norway; 2 Clinic of Surgery, St. Olavs Hospital, Trondheim University Hospital, Trondheim, Norway; 3 Central staff, St. Olavs Hospital HF, Trondheim, Norway; 4 Department of Pathology, St. Olavs Hospital, Trondheim University Hospital, Trondheim, Norway; 5 Department of Clinical and Molecular Medicine, NTNU - Norwegian University of Science and Technology, Trondheim, Norway; 6 Department of Radiology and Nuclear Medicine, St. Olavs Hospital, Trondheim University Hospital, Trondheim, Norway; 7 Central Norway Regional Health Authority, Stjørdal, Norway; 8 HUNT Center for Molecular and Clinical Epidemiology, Department of Public Health and Nursing, NTNU - Norwegian University of Science and Technology, Trondheim, Norway; 9 BioCore - Bioinformatics Core Facility, NTNU – Norwegian University of Science and Technology, Trondheim, Norway; 10 Clinic of Laboratory Medicine, St.Olavs Hospital, Trondheim University Hospital, Trondheim, Norway

**Keywords:** Prostate cancer, Tumour biomarkers, Tumour heterogeneity, Transcriptomics, Cancer microenvironment

## Abstract

Prostate tumor heterogeneity is a major obstacle when studying the biological mechanisms of molecular markers. Increased gene expression levels of secreted frizzled-related protein 4 (SFRP4) is a biomarker in aggressive prostate cancer. To understand how *SFRP4* relates to prostate cancer we performed comprehensive spatial and multiomics analysis of the same prostate cancer tissue samples. The experimental workflow included spatial transcriptomics, bulk transcriptomics, proteomics, DNA methylomics and tissue staining. *SFRP4* mRNA was predominantly located in cancer stroma, produced by fibroblasts and smooth muscle cells, and co-expressed with extracellular matrix components. We also confirmed that higher *SFRP4* gene expression is associated with cancer aggressiveness. Gene expression of *SFRP4* was affected by gene promotor methylation. Surprisingly, the high mRNA levels did not reflect SFRP4 protein levels, which was much lower. This study contributes previously unknown insights of *SFRP4* mRNA in the prostate tumor environment that potentially can improve diagnosis and treatment.

## Abstract (web summary)

Leveraging spatial and bulk transcriptomics, DNA methylomics and proteomics on the same prostate cancer tissue samples, this study provides insights on the tissue origin and role of SFRP4 gene expression in aggressive prostate cancer.

## Introduction

Prostate tissue is inherently heterogenous, and a given sample may contain a mix of cancer glands with different histological grade groups (GG), normal glands, stroma and lymphoid aggregates. This heterogeneity is poorly reflected in traditional molecular bulk methodology which results in averaged measurements. This poses a considerable challenge in understanding cancer biology since cells in the tumor microenvironment (TME) and their interplay with cancer cells are fundamental for prostate cancer progression^1^. Although cell culture-based research has led to impactful discoveries of cancer cell function, these experiments face major challenges in reproducing the true biological complexities between cancer and TME cells in vivo^2^.

One way to capture the true biological complexity is to measure a wide range of different molecules by combining multiple omics methodologies (i.e., multiomics). Spatially resolved omics-methods is especially effective to study heterogeneous cancer tissue. Spatial transcriptomics (ST) is a powerful method which allows for spatial mapping of mRNA molecules and has been applied to characterize both prostate cancer^3^ and other diseased tissues^4–7^. The spatial location of gene expression is important to uncover the biological functions of proposed oncogenes and biomarkers.

Secreted frizzled-related protein 4 (SFRP4) is a moderator of Wnt signaling, a pathway which promotes cell fate specification, proliferation and migration. Wnt signaling is crucial during embryonic development but is additionally identified as upregulated in several tumors. The SFRP4 protein is a suggested tumor suppressor thought to inhibit Wnt-signaling by hindering the extracellular Wnt-ligand to attach to the receptor^8,9^. However, we and others have identified increased *SFRP4* gene expression with increasing prostate cancer aggressiveness^10–14^, which also have been found for several other cancers^15–18^. In the context of personalized medicine, *SFRP4* gene expression has the potential to improve diagnosis and prognosis accuracy. The biological role of SFRP4 in heterogeneous tumors is still unknown and it is unclear which cells in the tissue are producing it. Previous immunohistochemistry (IHC) staining has indicated the SFRP4 protein to be located in the epithelial gland cells^11^. However, SFRP4 is a secreted protein and can therefore be synthesized by other cells in the tissue.

In this study, we investigated the biological role of SFRP4 in prostate cancer tissue by multiomics analysis of the same tissue samples, including ST, bulk transcriptomics, laser micro dissected (LMD)- and mass spectrometry (MS)-based proteomics, DNA methylomics, histopathology and Masson’s trichrome staining. The prognostic value of *SFRP4* gene expression were assessed using clinical follow-up data (>10 years). Several publicly available datasets, including transcriptomics, methylomics and single cell transcriptomics, were used to further investigate and validate the role of SFRP4 in prostate cancer.

## Results

### Multiomics profiling of SFRP4 in prostate cancer tissue

ST data was acquired with the Visium Spatial gene expression kit from 10× Genomic. With this technology circular spots of 55 µm distanced 200 µm from each other can capture hundreds of spatially defined gene expression profiles on a single tissue section. ST data of 32 prostate cancer tissue samples (*N* = 8 patients) allowed examination of the spatial distribution of *SFRP4*, a biomarker we previously have associated with cancer aggressiveness^10,19^. Five of the patients experienced relapse after radical prostatectomy, while three have no recorded relapse. Histopathology and GG were annotated by two uropathologists. We classified each ST spot in one of the following histology categories; non-cancer gland, low-grade (LG) cancer (defined as GG1 and GG2), high-grade (HG) cancer (GG3-5), lymphocytes, stroma normal, stroma LG cancer and stroma HG cancer. All stroma spots were classified based on which samples they resided in, e.g., stroma in samples with any HG cancer were classified as ‘stroma HG cancer’. See Supplementary Fig. 1 for spatial distribution of histology classes. The ST dataset contained 22,224 spots and 26,000 genes, which was reduced to 19,854 spots and 2435 genes after data processing and filtering.

**Fig. 1:**
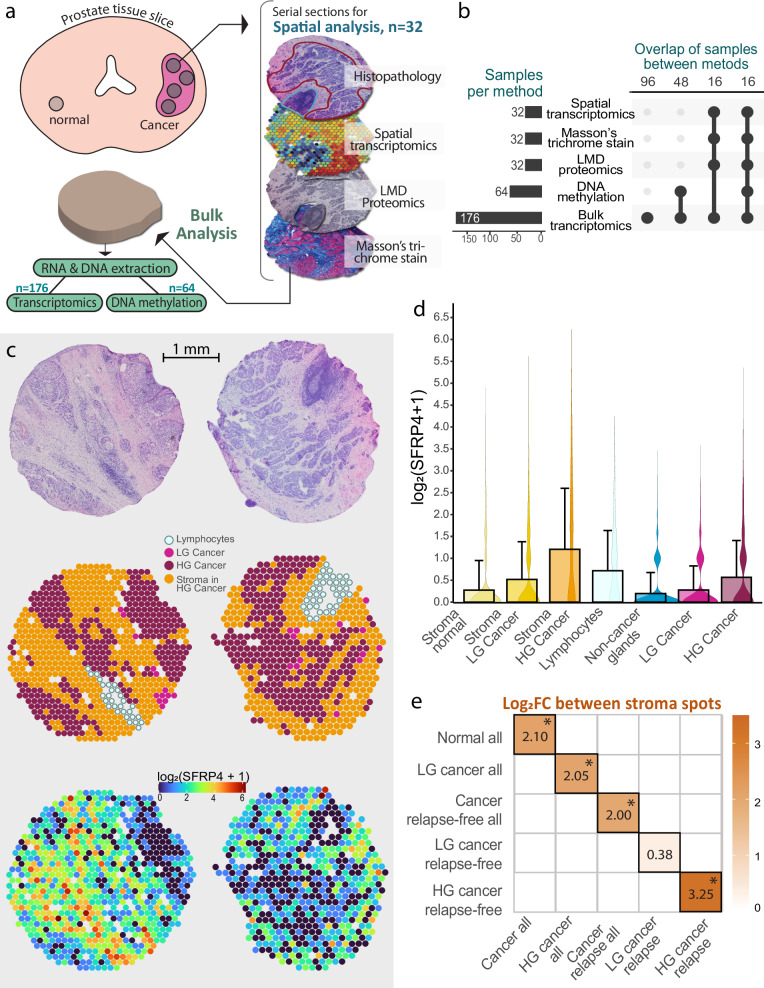
Spatial transcriptomics and multiomics analysis of *SFRP4.* **a** Workflow for collection of prostate cancer samples and multiomics analysis, which included both spatially resolved and bulk methodologies. **b** Overview of number of samples with different omics data and the overlap of samples between the different methodologies. **c** Spatial transcriptomics visualization of the two samples with the highest *SFRP4* expression, showing H&E image, histology classifications and *SFRP4* expression levels. **d** Bar plot of average gene counts (error bars show standard deviation) along with corresponding violin plots demonstrating the *SFRP4* expression levels across the different histology groups in the whole dataset (*n* = 19 854 spots). **e** Diagram showing log_2_ fold change (log_2_FC) after differential analysis with edgeR comparing stroma spots of aggressive groups (*x*-axis) to the less aggressive group (*y*-axis). Values illustrated in (**a**, **b**) are given as log_2_(count+1). LMD laser microdissection, HG high-grade, LG low-grade.

After ST, we performed LMD proteomics and Masson’s trichrome staining on serial sections and bulk transcriptomics and DNA methylomics on the remaining tissue (Fig. 1a). All ST samples were analyzed with bulk transcriptomics, LMD proteomics and Masson’s trichrome staining, while there was an overlap of 16 samples with the DNA methylomics analysis (Fig. 1b, Supplementary Data 1). A total of 176 samples from 37 patients (27 with relapse) were included in this study. Clinical follow-up data including pre-surgery prostate-specific antigen (PSA), T-stage and time to relapse after surgery are presented in Table 1 and Supplementary Table 1.

**Table 1:** Clinical parameters of patients

	Relapse	
	Yes (*n* = 27)	No (*n* = 10)
Clinical GG post-surgery		
2	7	8
3	10	2
4	4	0
5	6	0
Age at radical prostatectomy		
50–59	10	6
60–69	14	4
70–73	3	0
Preoperative PSA		
4.0–9.9	11	4
10.0–19.9	9	5
≥20.0	7	1
Clinical T-stage^a^		
T2c	9	9
T3a	6	0
T3b	12	0
Months from surgery to relapse		
≤ 12	15	
≤ 48	10	
> 48	2	
Omics analysis		
Spatial transcriptomics	5	3
Bulk transcriptomics	27	10
Bulk methylomics	10	6
Masson’s trichrome stain	5	3
LMD proteomics	5	3

Includes clinically recorded grade group (GG), T-stage after surgery, age at surgery, serum PSA before surgery and the number of months from surgery to relapse (for the patients with relapse). An overview of the number of patients with measurements for the different omics analysis is also presented.

^a^One relapse-free patient had unknown T-stage.

### Gene expression of *SFRP4* is predominantly located in stroma

ST demonstrated that *SFRP4* gene expression was predominantly located in the stroma of samples with HG prostate cancer (GG ≥ 3) (Fig. 1c, d, Supplementary Fig. 2). In contrast, previous IHC staining of the SFRP4 protein in prostate cancer samples predominantly show staining in epithelial cells^10–12^. However, as SFRP4 is a ligand functioning in the extracellular space, the production origin and the final location of SFRP4 may not be the same.

The *SFRP4* expression levels across all the different histology groups revealed that stroma of HG cancer samples had the highest levels of *SFRP4*, followed by, lymphocytes, HG cancer and stroma of LG spots (Fig. 1d). We noted that several areas with HG cancer had cancer cells that were highly mixed with stroma cells. This would explain the high levels of *SFRP4* expression in HG cancer spots (Fig. 1d).

### *SFRP4* gene expression is associated with prostate cancer aggressiveness

Differential expression (DE) analysis was performed to investigate how gene expression of stroma spots differ depending on cancer grade and patient relapse status (Supplementary Data 2). As presented in Fig. 1e, *SFRP4* gene expression had significantly increased fold changes in stroma cancer compared to stroma non-cancer spots (log_2_FC = 2.098, *p* = 2.18 × 10^−230^) and in stroma HG cancer compared to stroma LG cancer spots (log_2_FC = 2.048, *p* = 5.44 × 10^−175^). *SFRP4* was also increased in cancer stroma from relapse patients compared to relapse-free patients (log_2_FC = 1.97, *p* = 1.33 × 10^−119^). The increase in relapse patients was also true when only including HG cancer stroma (log_2_FC = 3.25, *p* = 3.59 × 10^−66^). *SFRP4* was additionally increased in LG cancer stroma from relapse patients compared to relapse-free patients (log_2_FC = 0.382) although this was not significant (*p* = 1.00). To conclude, *SFRP4* gene expression was associated with the more aggressive group in all comparisons (Fig. 1e), which aligns with our previous report for several different patient cohorts^10^.

### *SFRP4* gene expression is related to time to relapse

Bulk transcriptomics analysis was also performed on 176 tissue samples (*N* = 37 patients), including the 32 samples used for ST. For *SFRP4*, there was a correlation of 0.87 (*p* < 0.001) between bulk transcriptomics and ST (average per section) measurements, demonstrating high concordance. Given that *SFRP4* expression was observed to be higher in stroma spots (Fig. 1d), we explored whether *SFRP4* levels in bulk transcriptomics data varied with stroma content. Interestingly, both tissue samples high (≥65%) and tissue samples with low (≤35%) stroma content exhibited higher *SFRP4* levels compared to those with medium stroma content (36–64%) (Supplementary Fig. 3a). This indicates that while tissue composition is a confounding factor, *SFRP4* gene expression does not correlate linearly with the percentage of stroma present. Notably, samples with low stroma content (≤35%) were dominated by cancer samples (*n* = 71) and while very few non-cancer samples (*n* = 5) had low stroma content (Supplementary Fig. 3b). These findings suggest that tumor tissue maintains high *SFRP4* mRNA production even as stroma areas decrease.

**Fig. 2:**
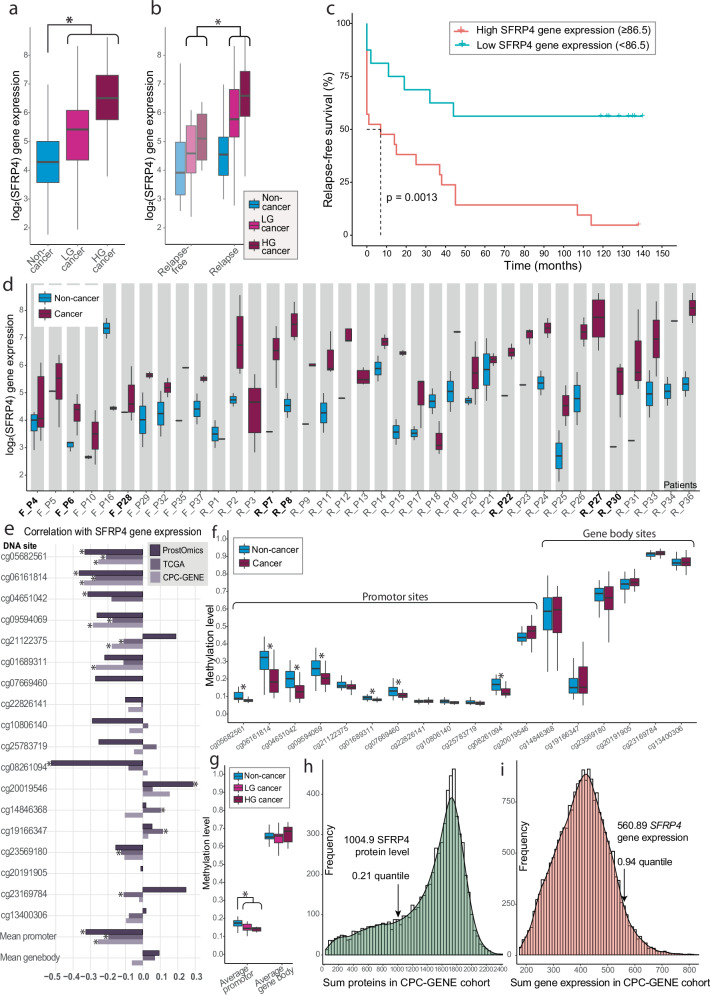
Multiomics results of SFRP4. Bulk transcriptomics; **a** Log_2_-transformed *SFRP4* gene expression was elevated from non-cancer (*n* = 61) to low-grade (LG) cancer (*n* = 61) and further in high-grade (HG) cancer samples (*n* = 54). **b** *SFRP4* gene expression levels were also higher in cancer samples from relapse patients compared to relapse-free patients. **c** Kaplan–Meier plot revealed *SFRP4* to be significantly associated with time to relapse for the 37 patients. The *SFRP4* cutoff value was determined by using the Cutoff Finder tool^67^. **d** *SFRP4* gene expression levels across all patients (*n* = 37). The ‘R’ and ‘F’ in front of the patient ID represent relapse (*N* = 27) and relapse-free patients (*N* = 10), respectively, while the patients where ST data are available are marked with bold text. DNA methylomics; **e** DNA promotor sites were significantly correlated with *SFRP4* gene expression across our cohort (ProstOmics, *n* = 64 samples) and the publicly available datasets TCGA-PRAD (The Cancer Genome Atlas Prostate Adenocarcinoma, *n* = 532) and CPC-GENE (The Canadian Prostate Cancer Genome Project *n* = 210). The *SFRP4* gene promotor was significantly hypomethylated in cancer (*n* = 35) compared to non-cancer samples (*n* = 29) as shown across (**f**) all 18 methylation sites and (**g**) mean of promotor and gene body sites. Proteomics; Histogram of (**h**) summed protein and (**i**) gene expression levels in the publicly available CPC-GENE cohort show a higher *SFRP4* mRNA level than SFRP4 protein relative to their respective datasets. The summed total and quantile levels of SFRP4 protein and gene expression levels are marked. Significance (*p* < 0.05) is symbolized with *.

Linear mixed models (LMM) DE with patient origin as random effect was used to test differences in *SFRP4* mRNA levels in the bulk transcriptomics data. Patient age had a slight, but significant correlation (Spearman *ρ* = 0.33, *p* = 0.046) with *SFRP4* levels and were therefore together with stroma content adjusted for in the LMM analysis. As shown in Fig. 2a and Supplementary Table 2, this analysis revealed significantly higher *SFRP4* mRNA levels in cancer compared to non-cancer samples (logFC = 1.59, *p* = 7.44 × 10^−14^). No significant difference was detected between HG and LG cancer samples (logFC = 0.32, *p* = 0.26). Comparing samples from relapse-free patients with relapse patients demonstrated significantly higher levels of *SFRP4* in the relapse patients (Fig. 2b, Supplementary Table 2) when including cancer samples (logFC = 1.13, *p* = 0.019), but not when including all samples (logFC = 0.87, *p* = 0.06). The Kaplan–Meier curve showed a significant association between high *SFRP4* gene expression and faster relapse (Fig. 2c). These findings align with both our results from ST (Fig. 1b) and our previous publication where elevated *SFRP4* gene expression was associated with a worse clinical outcome in seven different cohorts with in total 1404 patients^10^.

Since patient origin can influence gene expression^20^, we investigated the *SFRP4* levels between non-cancer and cancer samples within the same patient. Although there was patient-related gene expression variation, of the 34 patients where we had both cancer and non-cancer samples, the majority (*n* = 31, 91%) had higher *SFRP4* gene expression in the cancer samples (Fig. 2d).

### Reduced methylation of *SFRP4* promotor in cancer

Methylation of genes contribute to regulate gene expression and changed methylation of the *SFRP4* promotor has been observed for several forms of cancers^21^. We therefore investigated the *SFRP4* methylation patterns in our own data (64 samples, 16 patients). A total of 18 methylation sites mapped to the *SFRP4* gene (UCSC annotation), of which 12 were on the promotor region and six were in the gene body. Comparing the methylation levels to *SFRP4* gene expression demonstrated a significant negative correlation between the average promotor methylation and gene expression (Fig. 2e, *ρ* = −0.33, *p* = 0.041, Supplementary Table 3). There was no significant association between gene body methylation and gene expression of *SFRP4*. We also validated this finding in the publicly available datasets TCGA-PRAD (*n* = 532)^22^ and CGC-GENE (*n* = 210)^23^ which produced similar results although the correlation level was weaker for the average promotor region; *ρ* = −0.20 (*p* = 2.56 × 10^−5^) and *ρ* = −0.26 (*p* = 6.62 × 10^−4^), respectively (Fig. 2e, Supplementary Table 3). The cg06161814 promotor site had overall the strongest negative correlation (Fig. 2e).

Through LMM differential methylation analysis we found several differences in methylation when comparing non-cancer to cancer samples (Fig. 2f, g, Supplementary Table 4, Supplementary Fig. 4a). Seven out of 12 promotor sites were significantly hypomethylated in cancer compared to non-cancer samples, of which the cg06161814 site showed the largest and most significant change (*p* = 8.5 × 10^−5^, Fig. 2f). In contrast, there were no significant changes in DNA methylation between samples from relapse patients compared to relapse-free patients (Supplementary Table 4, Supplementary Fig. 4b). When comparing LG to HG cancer samples one site was significantly more methylated (cg20019546, *p* = 0.047) Supplementary Table 4). The gene body methylation levels were generally higher than the promotor region (Fig. 2g, Supplementary Fig. 4). This is not surprising as increased promotor methylation is more predictive of gene silencing and lower expression, while gene body methylation is associated with higher expression^24,25^.

### SFRP4 protein is low abundant in prostate tissue

We analyzed 114 LMD areas from serial sections of the same samples used for ST. Despite the detection of 5795 different proteins, the SFRP4 protein was not detected in any of the analyzed LMD areas (Supplementary Data 3). SFRP4 is a glycoprotein with several glycans attached. Glycan-modification changes the mass of tryptic peptides in an unpredictable way causing problems for MS/MS identification, which assumes glycan-free amino acid sequences. However, there are still several tryptic peptides without modifications that should result in identification if sufficient levels are present^26^. Our results therefore suggest that SFRP4 protein levels were under the limit of detection and are expressed at a substantially lower level than mRNA *SFRP4* in our prostate cancer samples.

To validate if SFRP4 protein levels typically are lower than gene expression, we used the publicly available prostate cancer CPC-GENE datasets^23,27^ which included proteomics (*n* = 76) and transcriptomics (*n* = 213) data, of which 63 samples had both omics available. Both datasets had SFRP4 detection. We summed all proteins (7054) and gene expressions (21,055) across all 63 samples to get an estimate of the relative levels of SFRP4 protein and *SFRP4* gene expression. The SFRP4 protein levels were low compared to other proteins in the dataset with levels in the low 21% percentile (Fig. 2h). In contrast, *SFRP4* gene expression were in the 94% percentile and thereby among the highest expressed genes (Fig. 2i). Spearman correlation between SFRP4 protein and gene expression levels (*n* = 47, excluding samples with no SFRP4 protein detection) had a non-significant and slightly inverse association (*r* = −0.21, *p* = 0.15). Clearly, there are substantial differences between gene expression and protein levels of SFRP4.

### *SFRP4* is associated with expression of ECM genes

**Fig. 3:**
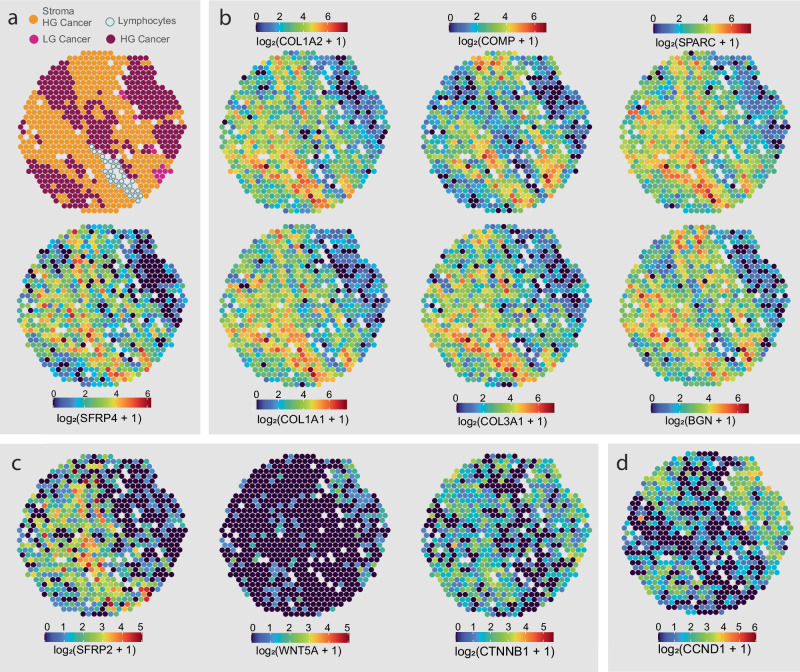
Spatial gene expression distribution of *SFRP4* and related genes. The spatial distribution of **a** *SFRP4* was similar and correlated with **b** genes transcribing extracellular matrix components, *COL1A1, COL1A2, COL3A1, SPARC, COMP* and *BGN*. **c** With the exception of *SFRP2, SFRP4* had no clear correlation with Wnt pathway genes such as *WNT5A* or *CTNNB1* (beta-catenine). **d** Wnt target gene *CCND1* showed higher levels in epithelium. *SFRP4* expression in stroma had a positive correlation with *CCND1* levels in epithelium of the same samples. All distribution images are from the same sample. Distribution of all samples for the presented genes can be viewed in Supplementary Figs. 5–14.

To explore the biological role of SFRP4, we correlated its gene expression to all other genes in the ST dataset. All top 6 (*R* > 0.4) positively correlated genes were prominent ECM components; collagen type I alpha chain 1 (*COL1A1*), *COL1A2, COL3A1*, secreted protein acidic and cysteine rich (*SPARC*), cartilage oligomeric matrix protein (*COMP*) and biglycan (*BGN*) (Supplementary Data 4, Supplementary Figs 5–10). This was also confirmed by visually comparing the spatial distributions (Fig. 3a, b). Interestingly, Mortensen et al. found that *SFRP4* gene expression clustered together with the same genes which was predictive of aggressive prostate cancer when compared to indolent cases^12^.

### Relating *SFRP4* to Wnt pathway-related gene expression

As SFRP4 is a modulator of the Wnt-pathway, we also explored the gene expression of other Wnt-pathway genes. Among the 33 Wnt genes assessed in our previous publication^19^, only 4 genes were sufficiently detected in the ST data. *Wnt5A* and beta-catenin (*CNNB1*) were located in the epithelium rather than the stroma and did not have a strong correlation with *SFRP4* (ranked 324 and 1437, Supplementary Data 3, Supplementary Figs. 11 and 12). The exception was *SFRP2*, which showed a similar distribution to *SFRP4* (Fig. 3c, Supplementary Fig. 13) and was among the genes with the highest association with *SFRP4* (ranked 33). This is likely explained by *SFRP2* having a similar function as an extracellular modulator of the Wnt-pathway^28^. Similarly to *SFRP4*, *SFRP2* gene expression was always higher in the more aggressive group compared (DE analysis of ST data, Supplementary Data 1).

As the Wnt pathway had the highest gene expression in the epithelium, we hypothesize that the Wnt pathway is more active in epithelial cells. SFRP4 produced in stroma could influence Wnt-pathway activity in adjacent epithelial cells, and lead to altered gene expression of Wnt-target genes. We investigated whether the epithelial expression of eight reported Wnt gene targets were associated with *SFRP4* stromal gene expression. Cyclin D1 (*CCND1*) was the only gene significantly correlated with *SFRP4* (*ρ* = 0.55, *p* = 0.011, Fig. 3d, Supplementary Data 3, Supplementary Fig. 13). However, since we cannot confirm the presence of the SFRP4 protein, it is challenging to conclude on whether the correlation of *CCND1* is connected to SFRP4 modulating the Wnt pathway.

### Single-cell data demonstrate that fibroblasts and smooth muscle cells express *SFRP4*

**Fig. 4:**
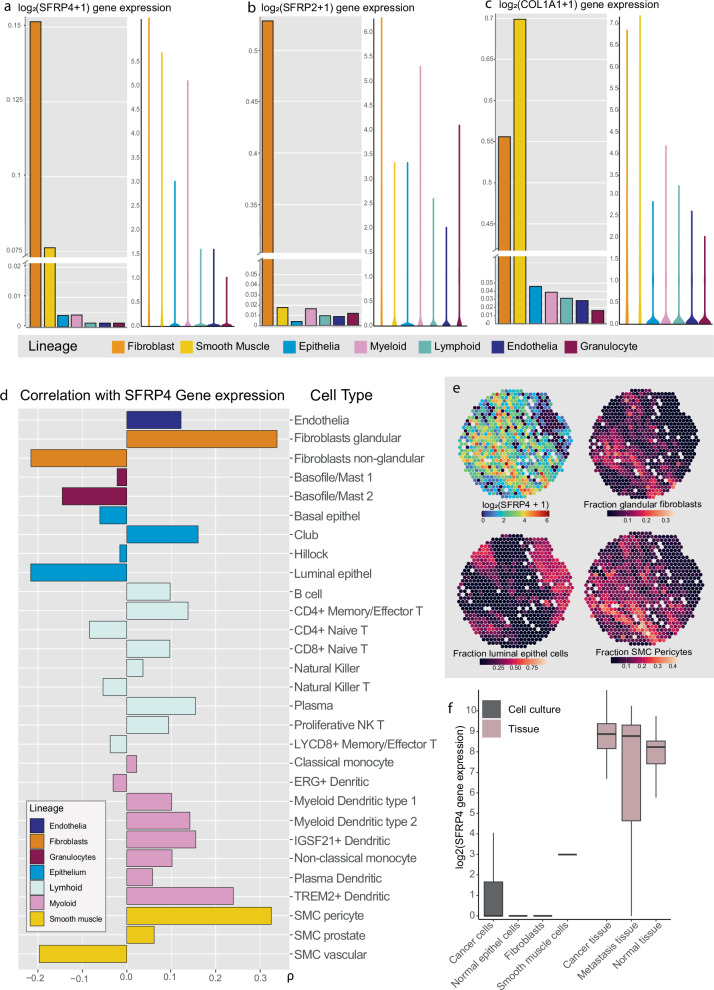
Relating *SFRP4* gene expression to cell type. Bar- and violin plots showing the gene expression for **a** *SFRP4*, **b** *SFRP2* and **c** *COL1A1* across the different cell lineages in the prostate single cell dataset (*n* = 83 451 cells)^30^. **d** Spearman correlation between *SFRP4* gene expression in our ST data and estimated cell type fractions (*n* = 19 854 spots). Bars are colored according to cell type lineage. **e** Spatial distribution of *SFRP4* gene expression shown together with estimated fractions of glandular fibroblasts, smooth muscle cell (SMC) pericytes and luminal epithelial cells. **f** *SFRP4* gene expression levels in the prostate tissue (pink) and in the cell culture (gray) dataset from Prensner et al.^33^.

To explore the cellular origin of *SFRP4*, we analyzed the publicly available single-cell transcriptomics dataset from the Strand lab^29,30^, generated from prostates of healthy organ donors and patients treated for benign prostatic hyperplasia. In this dataset the highest levels of *SFRP4* gene expression were found predominantly in fibroblasts, followed by smooth muscle cells (SMC), while *SFRP2* clearly had the highest gene expression level in fibroblasts (Fig. 4). The top 100 ranked genes correlated with *SFRP4* in the single-cell data included a high proportion of genes which products are either part of the ECM or regulate ECM remodeling (Supplementary Data 3). Similarly, to the ST data (Fig. 3b, Supplementary Data 3), this included *BGN, COMP, FN1, COL1A2, COL3A1* and several other collagens.

To assign cell type identity the Strand lab used key genes typically associated with specific cells^29,30^. By using the Stereoscope tool^31^, we estimated the cell type fractions for each ST spot. This allowed us to investigate which cell types *SFRP4* gene expression is the most associated with. As shown in Fig. 4d and Supplementary Data 3, *SFRP4* had the strongest correlation with glandular fibroblasts (*ρ* = 0.34) and SMC pericytes (*ρ* = 0.33). Glandular fibroblasts are adjacent to prostate epithelial glands^30^. If the stromal TME contributes to regulating the Wnt-pathway in epithelial cells, it would explain why the adjacent fibroblasts shows the highest *SFRP4* gene expression. Pericytes are most known for being a part of capillaries, but have a high expression of typical SMC proteins, including α-smooth muscle actin, vimentin, desmin, myosin, and nestin^32^. It is possible that, due to similar gene expression profiles, the fraction of pericytes is overestimated compared to prostate specific SMC. Nevertheless, pericytes are of the SMC lineage and our results does therefore indicate that SMC are producing *SFRP4* mRNA.

The strongest negative correlation with *SFRP4* gene expression was found for luminal epithelial cells (*ρ* = −0.22, Fig. 4d). While the estimated fractions of fibroblasts and SMC pericytes showed a similar distribution to *SFRP4* gene expression, fractions of luminal epithelial cells had an inverse spatial distribution (Fig. 4e, Supplementary Figs 15–17). This indicate that prostate epithelium is not the tissue compartment that produces *SFRP4*.

To validate that *SFRP4* is expressed by fibroblasts and SMC, we investigated the cell culture datasets from Prensner et al.^33^. The expression level of *SFRP4* was either barely detectable or not detected in the cell cultures (Fig. 4f). This was in contrast to the corresponding tissue data from Prensner which showed decent expression levels of *SFRP4* for normal, cancer and metastatic prostate tissue. This indicate that *SFRP4* expression is dependent on the interaction between different cells in a tissue environment. Cell cultures may have considerable limitations when investigating the in vivo role of *SFRP4* in prostate cancer progression.

### High *SFRP4* gene expression is associated with collagenous fibrosis

**Fig. 5:**
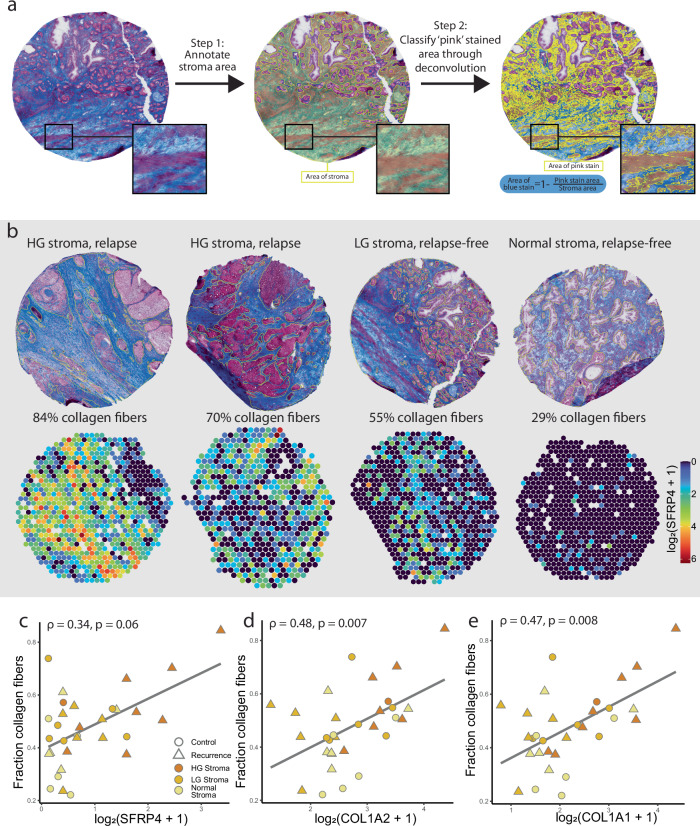
Spatial *SFRP4* gene expression compared to Masson’s trichrome stained serial sections. **a** Serial sections stained with Masson’s trichrome where first annotated for stroma areas (yellow). Within the stroma, areas with pink stain were segmented using deconvolution, and fraction of blue stain (collagen fibers) were calculated as the inverse of the pink stroma fraction. **b** Samples with varying collagen fiber fraction shown next to *SFRP4* ST gene expression. Collagen content was correlated with average gene expression of all stroma spots of each sample. The mean mRNA levels of **c** *SFRP4*, **d** *COL1A2* and **e** *COL1A1* were correlated and plotted against fraction of collagenous fibers in stroma (*n* = 31). The linear regression line is shown, and the color and shape of the data points indicates stroma type (normal, low-grade (LG) and high-grade (HG) stroma) and relapse status, respectively. Spearman correlation coefficient *ρ* and *p*-value are presented for (**c**–**e**).

Based on the association between *SFRP4* and ECM genes, we hypothesized that *SFRP4* could be connected to ECM remodeling or fibrosis. We therefore performed Masson’s trichrome staining on serial tissue sections (Supplementary Fig. 18) and estimated the collagen content by quantifying the fraction of blue stain in stroma (Fig. 5a). Estimated collagen fiber content ranged from 22% to 84% for the 31 samples examined (one excluded due to poor quality, Supplementary Table 6, Fig. 5b). When comparing the average *SFRP4* levels in stroma of each sample to the collagenous fiber content, there was a moderate and almost significant correlation (Spearman *ρ* = 0.34, *p* = 0.06). The samples with high *SFRP4* gene expression (log_2_ > 1 mean *SFRP4* gene count per stroma spot) did tend to have high corresponding fiber content (Fig. 5c). However, conversely, a high collagenous fiber content was not necessarily indicative of high *SFRP4* levels. A collagen rich ECM may therefore not be solely dependent on the presence of *SFRP4*. For the collagen genes *COL1A1* and *COLA2* there were stronger and more significant correlations to fibers content (*ρ* ≥ 0.47, *p* ≤ 0.008, Fig. 5d, e). As the Masson’s stain attaches to collagen, it is reasonable that the major collagen genes had a higher association than *SFRP4*.

## Discussion

SFRP4 has been extensively studied in the context of the Wnt-pathway and cancer development^34^. The mechanistic role of SFRP4 has been predominantly studied in cell culture^9,35,36^. However, the interactions between heterogenous groups of cells in tissue is far more complex than what can be reproduced in cell cultures. For this reason, we investigated the role of SFRP4 by using spatial and multiomics analysis of prostate cancer tissue. ST showed that *SFRP4* gene expression was predominantly located in the stroma of prostate cancer samples. We found through multiomics analysis that *SFRP4* mRNA levels seem to be partly regulated by gene promotor methylation, while *SFRP4* gene expression did not reflect SFRP4 protein abundance. Cell-type estimation with Stereoscope revealed that *SFRP4* is mostly expressed by stromal fibroblasts and SMC. Further, *SFRP4* gene expression was highly associated with markers for ECM remodeling. *SFRP4* is therefore likely a key player in the TME.

Both the spatial and bulk transcriptomics analysis showed that increased *SFRP4* mRNA levels were connected to prostate cancer aggressiveness. Wissman et al.^37^ were the first to report in 2003 increased *SFRP4* gene expression in prostate cancer tissue. Since then, this connection of *SFRP4* and aggressive prostate cancer has been confirmed by several other studies^9,12,19^. We previously found that increased *SFRP4* is predictive of prostate cancer aggressiveness in seven independent datasets, compromising 1404 individuals^10^. Using *SFRP4* gene expression as a clinical biomarker may still not be forthright as accuracy is confounded by heterogeneous tissue composition. Since *SFRP4* has a higher expression in the tumor stroma, high-grade cancer samples where cancer cells have displaced the stroma tissue might not consistently have high *SFRP4* gene expression in average bulk analysis. While many cancerous samples had a high *SFRP4* expression despite reduced stroma areas in our bulk transcriptomics dataset, the highest expression was still observed in HG-cancer samples with high stroma content (≥65%, Supplementary Fig. 3b). This demonstrates that *SFRP4* gene expression can be confounded by stroma content which complicates the clinical utility of *SFRP4* gene expression. A more likely robust clinical tool would be to use *SFRP4* as part of a gene signature by combining it with the expression of genes altered in the epithelial cancer cells. This strategy would minimize the risk of false negative test results due to low stroma content. *SFRP4* is already used as a part of several gene signatures that are predictive of a worse prostate cancer prognosis^14,38^, including the commercially available Oncotype Dx test^13,39^. The Oncotype Dx test calculates a gene signature score through taking tissue composition into account by combining markers for both prostate cancer cells and tumor stroma, which includes *SFRP4*. This demonstrates that even though the evidence connecting *SFRP4* mRNA levels to cancer aggressiveness is substantial, tissue composition needs to be accounted for when using *SFRP4* as a clinical biomarker.

DNA methylation, especially in the gene promotor region, are well known to influence transcription. We found that methylation of the *SFRP4* gene promotor was both significantly negatively correlated with gene expression and reduced in cancer compared to normal samples. Although there are several studies presenting genome wide methylation analysis of prostate cancer tissue^22,40–43^, few studies report on methylation of *SFRP4*. One study performed targeted methylation assays, but could not find any difference in methylation of the *SFRP4* promotor when comparing normal, BPH and prostate cancer samples^44^. To the best of our knowledge, no other publication has reported reduced *SFRP4* promotor methylation in prostate cancer compared to normal samples. Although we identified significant DNA methylation changes, it does not explain all *SFRP4* gene expression variation, which could be influenced by other processes such as histone modifications and transcription factor activity.

Notably, protein levels of SFRP4 did not match *SFRP4* gene expression. We could not detect any SFRP4 proteins in our LMD proteomics data. Additionally, the protein levels were substantially lower than the *SFRP4* mRNA levels in the CPC-GENE cohort (Fig. 2h, i). The original CPC-GENE proteomics publication found that gene expression could only account for 10% of the variation in protein levels^27^. They also observed that the low abundant proteins, such as SFRP4, had the weakest correlation with gene expression. There are potentially regulatory mechanisms causing the discrepancy between protein and gene expression levels in prostate tissue. Either the mRNA are not efficiently translated or SFRP4 proteins are readily degraded. The SFRP4 protein may be subjected to phagocytosis by macrophages, as reported for wound healing^45^. The low MS-detection of SFRP4 may also explain why we in a previous study had low IHC staining intensity, while *SFRP4* gene expression had robust levels^10^. This is also reflected in the staining intensities on prostate cancer tissue in the Protein Atlas database^46^. It should be noted that some studies^11,12^ have found associations between higher SFRP4 staining intensity with worse clinical outcomes. Others have in contrast reported reduced staining levels with increased prostate cancer aggressiveness^9,47^. Horvath et al. found that despite higher *SFRP4* mRNA in prostate cancer tissue, high IHC stain of SFRP4 protein was associated an improved prognosis^9^. Our results, along with the findings by others, suggest that the *SFRP4* gene expression is a suitable biomarker candidate, while SFRP4 protein levels show little promise as a clinical biomarker.

The ST analysis demonstrated that *SFRP4* is predominately expressed by cells in the prostate stroma, which is in agreement with previous studies^30,48^. Interestingly, *SFRP4* also had elevated levels in samples with high compared to low reactive stroma content in our previous study (log_2_FC = 0.92, Supplementary data of Andersen et al.^49^). A transcriptomics study using LMD found, similarly to us, that *SFRP4* had one of the highest increases when comparing stroma in HG cancer to stroma in LG prostate cancer samples^48^. The single cell transcriptomics dataset^30^ and cell-type estimation of our ST data showed that most of the *SFRP4* mRNA are found in fibroblasts followed by SMC. According to the Human Proteome Atlas^50^, *SFRP4* gene expression is the most enriched in fibroblasts across several human tissues^46^. Further, we found that *SFRP4* had the highest co-expression with genes involved ECM remodeling, such as *COL1A1, SPARC, COMP* and *BGN*. *SFRP4* gene expression also had a weak association with stroma fibrosis as determined from Masson’s trichrome stains (Fig. 5). Fibroblasts are known as the front-runners of ECM remodeling and fibrosis in tissues^51^. However, in stroma tissue SMCs are also reported to produce ECM components^52^. It is unclear how *SFRP4* gene expression by fibroblasts and SMC are associated with ECM remodeling.

The consensus has been that SFRP4 is a tumor suppressor by inhibiting the oncogenic Wnt-pathway^53^. However, we found no correlation between *SFRP4* and other Wnt-pathway genes. This is not surprising considering that SFRP4 function in the extracellular space and could be synthesized by other tissue areas than where the Wnt-pathway is activated. The Wnt-pathway is reported to be more activated in the epithelial tissue, to be a regulator of cell regeneration and is also therefore reported as an oncogenic pathway in epithelial tumors^54^. It is still unclear how *SFRP4* gene expression is related to the Wnt-signaling in tissue. A cancer biomarker can either be a driver or a passenger of cancer development^55^. It is possible that elevated *SFRP4* mRNA is merely a consequence (i.e., passenger) rather than a driver of prostate cancer. Only molecular drivers of cancer would be suitable drug targets. Understanding the true role of *SFRP4* mRNA and whether it would be a suitable drug target requires more research before reaching any conclusion.

While our study provides significant insights, certain limitations should be considered when evaluating the findings of this study. First, our patient cohort had a small sample size (*N* = 37) and low ethnical diversity. Cancer-associated gene expression can be influenced by ethnicity and can therefore be considered a confounding factor in transcriptomics studies^56^. Although ethnicity was not an available parameter for our data, the vast majority of these Norwegian patients are likely white. We can therefore not conclude on whether our observed associations between *SFRP4* levels and stroma in cancer samples are generalizable across all ethnicities. Further, despite the valuable power of spatial multiomics methods to provide insights of heterogeneous cancer tissue, spatial methodologies are limited by lower detection rates compared to bulk analysis. For instance, only 4 of 33 Wnt genes could be spatially investigated, making it challenging to get the full picture of SFRP4 in relation to the Wnt pathway. The detection sparsity also was apparent in our LMD proteomics data where the protein of interest, SFRP4, could not be detected at all. The protein abundance of SFRP4 is a major part of the puzzle and the absence of detection pose a clear limitation of this study in achieving a full understanding of the biological mechanism of SFRP4. Furthermore, despite the clear advantage of multiomics analysis of human tissue samples, this approach can only provide molecular snapshots. This is a limitation of our study for achieving a comprehensive functional understanding of cellular mechanisms related to SFRP4. Although, cell cultures are popular experiments to study molecular function, they fail to reflect the complex interactions between different cell types in heterogeneous tumor tissues. This is particularly true when it comes to SFRP4 where there is a mismatch between cell culture and human tumor tissues. This discrepancy is demonstrated by Garcia-Tobilla et al. who detected significantly higher *SFRP4* gene expression in normal prostatic cell lines compared to cancer cells, but found a completely reversed trend when analyzing tissue samples^44^. Decoding the true functions of SFRP4 in prostate cancer progression has proven to be challenging as demonstrated by both this and other studies.

To conclude, we investigated the biological context of the *SFRP4* biomarker by using comprehensive spatial and multiomics analysis of heterogenous prostate cancer tissue, an approach not previously applied to analyze SFRP4. Our findings show that *SFRP4* gene expression is predominantly located in the stroma of high-grade cancer samples and is tightly linked to ECM remodeling. The lack of association between *SFRP4* mRNA and protein levels raises important unanswered questions regarding biological mechanisms and indicates that only *SFRP4* gene expression holds strong potential as a biomarker. Further, we found that *SFRP4* levels is partly determined by methylation of the promotor region, though other mechanisms are likely also at play. Our survival analysis adds further weight to the evidence linking *SFRP4* to a poorer clinical prognosis and our spatial investigation emphasizes the critical need to account for tissue composition.

## Methods

### Patient inclusion and sample collection

All human prostate tissue material used in this study were collected after informed written consent was given by prostate cancer patients undergoing radical prostatectomy. This research was approved by the regional ethical committee of Central Norway (identifier 2017/576), and all methods were performed according to national and EU ethical regulations. All ethical regulations relevant to human research participants were followed.

Samples from eight patients diagnosed with prostate cancer and treated by radical prostatectomy at St. Olav’s hospital, Trondheim in the period 2008–2016 were included in this study. We selected three relapse-free patients (no confirmed relapse after 12–13 years) and five patients who experienced relapse with confirmed metastasis within three years after surgery. None of the patients received treatment prior to surgery. Immediately after surgical removal, a 2 mm thick slice was cut from the middle of the prostate (transverse plane), snap frozen and stored at −80 °C as described by Bertilsson et al.^57^. Tissue slice collection after surgery and all storage were facilitated by expert personnel at Biobank1®, St Olavs University Hospital, Trondheim, Norway. A range of 8–13 tissue cores (3 mm in diameter) were later collected fresh frozen from each tissue slice, using an in-house built drill system. Four samples were selected from each patient based on histopathology evaluation of hematoxylin erythrosin saffron stained tissue sections. For each whole-mount slice, we aimed for two samples with cancer tissue, one sample with non-cancer morphology close to cancer tissue and one sample with non-cancer morphology far away from the cancer area.

### Cryosectioning

All sections used in this study were 10 µm thick and cut on a cryostat (Cryostar NX70, Thermo Fisher Scientific) at −20 °C. The sections used for ST were placed within the 6.5 × 6.5 mm capture areas on Visium slides provided in the the Visium Spatial Gene Expression Slide Kit (art.nr PN-1000185, 10× Genomics) and stored at −80 °C until further processing. A total of 21 serial sections were collected from each sample and placed on different types of slides (super frost, conductive slides, membrane slides for LMD). These sections were collected for other and future methodologies, many of which are not included in this study.

### Preparing spatial sequencing libraries

Sequencing libraries were created from the tissue sections by using the Visium Spatial Gene Expression Slide & Reagent kit (art.nr PN-1000184, 10× Genomics) following the manufacturers manual. In brief, tissue sections were fixed using methanol, followed by H&E staining and immediately scanning of slides at ×20 magnification. A coverslip was temporary used over the sections for the microscopic scanning and then gently removed after. To capture mRNA, the tissue sections were incubated with permeabilization enzyme for 12 min, which previously had been optimized using the Visium Spatial Tissue Optimization Slide & Reagent Kit (art.nr PN-1000193, 10× Genomics). A second strand mix was added to create a second strand, and hereafter cDNA was amplified by real time qPCR. The amplified cDNA library was quantified with qPCR using the QuantStudio™ 5 Real-Time PCR System (art.no A34322, Thermo Fisher) and the cDNA libraries were stored at −20 °C until further use. Paired-end sequencing was performed on an Illumina NextSeq 500 instrument (SY-415-1001, Illumina®, San Diego, USA) using the NextSeq 500/550 High Output kit v2.5 (150 cycles) (art.no 20024907).

### Bulk RNA and DNA isolation

After cryosectioning for ST and other downstream spatial analysis, the remaining tissue material was used for RNA and DNA isolation. The Allprep® DNA/RNA/miRNA Universal Kit (art.nr 80224, QIAGEN, Hilden, Germany) was applied according to manufacturer’s protocol using mean input material of 7.69 mg (range 4.76–13.05 mg). Concentration of isolated RNA and DNA was quantified using a Qubit 3.0 Fluorometer (art.no Q33216, Thermo Fisher) with the Qubit RNA BR Assay Kit (art.no Q10211) and Qubit dsDNA BR Assay Kit (art.no Q32853), respectively. Isolated material was stored at −80 °C until further use.

### Bulk RNA-seq and DNA methylation analysis

For creating the cDNA library, the SENSE mRNA-Seq Library Prep Kit V2 (art.no 001, Lexogen, Vienna, Austria) was used according to the provided user manual using 600 ng RNA as input. The final cDNA library was quantified using Caliper GX (art.no CLS151164, Perkin Elmer) with the DNA High Sensitivity assay, and stored at −20 °C until further use. Single-read sequencing was performed on an Illumina NextSeq 500 instrument (Illumina®) using the NextSeq 500/550 High Output kit v2.5 (75 cycles) (art.nr 20024906).

For DNA methylation analysis, the EZ DNA Methylation™ Kit (art.no D5001, Zymo Research, Orange, USA) was used for bisulfite conversion using 1 µg DNA as input, followed by using the Illumina Human Metylation EPIC BeadChip kit (art.no WQ-317-1001, Illumina) as the DNA methylation assay. A total of 64 prostate tissue samples were analyzed, of which 16 samples also had been used for ST.

### Proteomics

LMD tissue section areas were analyzed with liquid-chromatography tandem mass spectrometry (LC-MSMS) for microproteomics analysis (see Supplementary Method). This was performed on serial sections of the same 32 samples analyzed with ST. The section used for LMD proteomics had a distance to the ST section ranging from 20 to 80 µm.

### Histopathology

The digital H&E scans were independently evaluated and annotated by two experienced uropathologists (T.V. and Ø.S.) using QuPath version (v 0.2.3). Different tissue types that were annotated by the pathologists were lymphocytes and cancer areas graded according to the Grade Group (GG) system^58^, in addition to glands of uncertain cancer status. Different areas of the tissue section were separated based on pure Gleason pattern; i.e., a sample with well-defined separate areas of Gleason 3 and 4 were annotated as separate regions of GG 1 and 4, respectively, rather than one GG 2 or 3 (Gleason 3 + 4 or 4 + 3) region. Additionally, tissue borders, lumen, stroma and epithelial areas were annotated in QuPath.

For further supervised data analysis, a consensus pathology evaluation was reached in agreement with both pathologists. For each ST spot, the fraction of different tissue types and regions present were calculated. Briefly, binary images for all annotation classes (stroma, tissue fold, epithelium etc.) were exported from QuPath using an in-house developed groovy script and merged with the ST data using our own python package^59^. The overlap of the binary images and spots was used to produce percentage values for each annotation class for each spot. These percentage values were used to assign each spot to one histopathology class. Spots that were either >50% outside the tissue border, had >50% tissue fold, >80% luminal space or >50% uncertain area were excluded. Spots with >55% stroma were assigned as stroma, if not the spot was assigned as the dominating annotation class i.e., either as lymphocytes, non-cancer epithelium or cancer with GG range 1–5. Cancer spots were assigned as low-grade (GG 1 and 2) and high-grade (GG 3–5). For the purpose of this study, stroma spots were further classified as ‘normal stroma’, ‘low-grade cancer stroma’ and ‘high-grade cancer stroma’ depending on whether the rest of the sample contain only non-cancer glands, had some low-grade (LG) prostate cancer (GG 1 and 2, overall sample score) or high-grade (HG) prostate cancer (GG 3–5, overall sample score), respectively. Spatial overview of histology class assignments for all samples are presented in Supplementary Fig. 1.

For further characterization of the stroma tissue, a separate set of serial sections from the same samples as used for ST, were stained with Masson’s trichrome staining, which stains collagen fibers blue and muscle fibers pink. The section used for Masson’s trichrome staining had a distance to the ST section ranging from 60 to 160 µm. After staining the sections were digitally scanned and imported into QuPath (v0.3.2), where the stroma area of each sample was annotated. Optimal stain deconvolution vectors were found to be (0.891, 0.454, 0.001) for the blue stain and (0.245, 0.931, 0.271) for the pink stain and was applied for all sections. Within the stroma, each pixel was then classified as ‘pink’ (i.e., muscle) through thresholding. Resolution was set to 0.55 µm/pixel, Gaussian prefilter was used and a threshold in the range of 0.15–0.30 was applied based on staining intensity (Supplementary Table 6). The area of the new pixel classification annotation was divided by the total area of the stroma to get the fraction of ‘pink’ stain, and the inverse fraction were defined as ‘blue’ and fibrous. One Masson’s trichrome stained section (P07_6) was excluded due to poor quality.

### Data preprocessing

The Illumina sequencer generated raw data as base call (BCL) files for each sample analyzed with ST. The BCL files were converted to FASTQ files with the 10× genomics space ranger software package (version 1.0.0) according to manufacturer’s recommendations. The human reference transcriptome GRCh38 (version 3.0.0) was used to map sequences to genes. This first processing resulted in a large table of raw gene counts for each spot. Genes with less than 10 reads in less than 10 spots were filtered out.

To account for differing cell numbers present in each spot, the ST dataset was normalized based on cell count. The number of cells present for each spot was estimated using the nuclei detection feature in QuPath. Data with detected nuclei were exported from QuPath and imported into python where an in-house script and package was used to give cell count values for each spot^59^. Cell count normalization was performed by first dividing all the gene counts for each spot by the cell count of that spot. Next, all gene counts were multiplied by the median cell count number (21) and rounded to the nearest integer. All spots with less than 40 detected genes and/or 100 normalized total gene counts were excluded.

FASTQ files from bulk transcriptomics analysis were filtered and trimmed using fastp v0.20.0. For sequence alignment the STAR tool was used^60^ against a reference set (Ensembl, GRCh38 release 92). Subsequently, featureCounts^61^ was used to extract gene counts from sequence reads according to the same reference set. Raw methylation data was normalized using the method described by Touleimat and Tost^62^ available in the *minfi* package for R. Methylation sites associated with SFRP4 were identified by the annotation of the probes available in the R package *IlluminaHumanMethylation-EPICanno.ilm10b4.hg19*. Gene feature groups TSS1500, TSS200, 5’UTR and 1stExon were defined as the ‘promotor region’ while 3’UTR and gene body were defined as the ‘gene body region’.

### Publicly available datasets

A number of publicly available datasets were downloaded and analyzed to study the role of *SFRP4* gene expression and other related genes. A single-cell transcriptomics dataset generated from healthy and hyperplastic human prostates^30^ containing data from 83,451 cells were downloaded from cellxgene.cziscience.com. Genes with less than at least 5 counts in at least 40 cells were filtered away, leaving 3840 genes. The microarray dataset from Prensner et al.^33^ which contained bulk transcriptomics data from human tissue (normal, cancer and malignant prostate) were downloaded from dbGaP (accession number phs000443.v1.p1) while cell culture transcriptomics data was retrieved from GEO (accession number GSE31728). Two publicly available datasets, TCGA-PRAD^22^ and CPC-GENE^23^, contained both mRNA expression and DNA methylation data and were downloaded from the Genomic Data Commons Data Portal (Accession number phs000178, https://portal.gdc.cancer.gov/projects/TCGA-PRAD) and the The Canadian Prostate Cancer Genome Project International Cancer Genome Consortium Data Portal (https://dcc.icgc.org/projects/PRAD-CA)^63^. TCGA-PRAD (*n* = 532 samples) is an RNA-seq dataset and CPC-GENE (*n* = 210) used microarray technology, and methylation analysis for both datasets were array-based. The CPC-GENE also has generated proteomics data which was retrieved from the supplementary data of the original publication^27^.

### Statistics and reproducibility

Differential expression analysis of the ST-data was performed using R-package *edgeR*^64^. For each pairwise comparison, a quasi-likelihood negative binomial generalized log-linear model was created. Each spot was considered as a ‘sample’ and spots categorized into the same group were considered biological replicates. An overview of the differential analysis tests performed, the groups compared, and sample sizes is presented in Table 2. As including a very large sample size (in this case up to 19,854 spots) tend to create very low *p*-values, even for genes with very small changes. Genes were therefore tested for whether they had significantly higher absolute log_2_ fold change (log_2_FC) than 0.5 (glmTreat-function). A Benjamini-Hochberg adjusted *p*-value higher than 0.01 was considered significant. Associations between the expression of different genes were analyzed using Spearman correlation in R.

**Table 2:** Overview of pairwise differential analysis of spatial transcriptomics (ST), bulk transcriptomics (BT) and bulk methylation (BM) data

Statistics	Data	Sample type	Variables	Group 1	Group 2
Quasi-likelihood negative binomial generalized log-linear model	ST	Spot	2436	Normal stroma (*n* = 3186)	Cancer stroma (*n* = 4802)
Quasi-likelihood negative binomial generalized log-linear model	ST	Spot	2436	LG Cancer stroma (*n* = 2620)	HG Cancer stroma (*n* = 2182)
Quasi-likelihood negative binomial generalized log-linear model	ST	Spot	2436	Relapse-free cancer stroma (*n* = 1469)	Relapse cancer stroma (*n* = 3333)
Quasi-likelihood negative binomial generalized log-linear model	ST	Spot	2436	Relapse-free LG cancer stroma (*n* = 1219)	Relapse LG cancer stroma (*n* = 1401)
Quasi-likelihood negative binomial generalized log-linear model	ST	Spot	2436	Relapse-free HG cancer stroma (*n* = 250)	Relapse HG cancer stroma (*n* = 1932)
Linear mixed models	BT	Bulk tissue	1	Non-cancer (*n* = 61)	Cancer (*n* = 115)
Linear mixed models	BT	Bulk tissue	1	Relapse-free (*n* = 49)	Relapse (*n* = 127)
Linear mixed models	BT	Bulk tissue	1	LG cancer (*n* = 61)	HG cancer (*n* = 54)
Linear mixed models	BT	Bulk tissue	1	Relapse-free cancer (*n* = 29)	Relapse cancer (*n* = 86)
Linear mixed models	BM	Bulk tissue	20	Non-cancer (*n* = 29)	Cancer (*n* = 35)
Linear mixed models	BM	Bulk tissue	20	Relapse-free (*n* = 20)	Relapse (*n* = 44)
Linear mixed models	BM	Bulk tissue	20	LG cancer (*n* = 17)	HG cancer (*n* = 10)
Linear mixed models	BM	Bulk tissue	20	Relapse-free cancer (*n* = 14)	Relapse cancer (*n* = 32)

For each statistical test the input sample was either a ST spot (spatial data) or whole tissue sample (bulk data). Samples within the same group being tested against another group were considered biological replicates.

*LG* low-grade, *HG*, high-grade.

For each ST spot we used the Stereoscope tool^31^ to estimate cell type fractions through deconvolution. By using a scanpy implementation of the Seurat method for highly variable gene identification and the publicly available singe-cell transcriptomics dataset from Joseph et al.^30^ as a reference dataset, we ended up with a list of 4324 highly variable genes. Stereoscope was run with this gene list and the following parameters: sc epochs = 75,000, sc batch size = 100, st epochs = 75,000, st batch size = 100, learning rate = 0.01.

To test if *SFRP4* was differentially expressed and if the *SFRP4* DNA methylation sites were differentially methylated in the bulk data, we created LMM with patient origin as random effect and adjusting for stroma content and patient age. Other potentially co-variating parameters such as PSA, grade group and T-stage are measures of cancer aggressiveness and were not controlled for as removing their effect also will remove a lot of the true effect of *SFRP4* gene expression on cancer aggressiveness. Stroma content and age were transformed from continuous into categorical variables; stroma content was divided into “≤35%”, “36–64%” and “≥65%”, while age was divided into “Age 51–58”, “Age 59–64” and “Age 65–73”. The *limma* R-package^65^ was used for the array methylation data (*n* = 64), while for the RNA-seq data (*n* = 176) the *edgeR* package^64^ was used. Prior to LMM, RNA-seq data were normalized to library size using the inbuilt function in egdeR. Through empirical Bayes moderated t-statistics four different comparisons were carried out for both the transcriptomics and methylation dataset; non-cancer vs cancer samples, relapse-free patient vs relapse patient samples, low-grade (GG 1 and GG 2) vs high-grade samples (GG 3–5) and relapse-free patient cancer vs relapse patient cancer samples (Table 2). For the *SFRP4* methylation data p-values were adjusted for multiple testing using the Benjamini-Hochberg method, and we used a significance threshold of *p* < 0.05.

The association between *SFRP4* bulk gene expression and relapse-free survival was tested using the *survival* package in R^66^. As there are multiple samples per patient (*N* = 37), the sample with the highest *SFRP4* gene expression was chosen to represent each patient. Prior to Kaplan–Meier analysis, the optimal *SFRP4* cutoff value was identified as 86.5 by using the Cutoff Finder tool^67^. A Kaplan–Meier curve was computed with time to relapse (*N* = 27 patients) after radical prostatectomy. Relapse was defined as either detectable PSA levels, spread of cancer cells to lymph nodes or metastasis.

### Reporting summary

Further information on research design is available in the Nature Portfolio Reporting Summary linked to this article.

## Supplementary information

Supplementary Information

Description of Additional Supplementary Files

Supplementary Data 1

Supplementary Data 2

Supplementary Data 3

Supplementary Data 4

Supplementary Data 5

Reporting summary

## Data Availability

The data generated and analyzed in this study includes sensitive information, and its management must comply with the General Data Protection Regulation (GDPR), Norwegian law, and the specific patient consent and ethical approval. Consequently, the data is legally subjected to restricted access. Raw and processed transcriptomics and DNA methylation data have been deposited at Federated European Genome-Phenome Archive (FEGA) Norway and are findable on the EGA portal (ega-archive.org) under the study ID EGAS50000000413. The spatial transcriptomics, bulk transcriptomics and DNA methylation data are deposited as separate datasets with the accession numbers EGAD50000000603, EGAD50000000604 and EGAD50000000605, respectively. Data access can be requested through the EGA portal, where any data request will be processed through a data access committee at NTNU. The proteomics data is not externally archived as there are currently no suitable public data repository that accept sensitive proteomics data and that meets the data sharing criteria postulated by the study’s ethical approval, patient consent, GDPR and Norwegian law. The proteomics data can be requested via email to maria.k.andersen@ntnu.no and may-britt.tessem@ntnu.no. For both archived and non-archived data, access will only be granted after the following steps have been achieved; 1. the data requester and the intended use of the data must comply with GDPR regulation, Norwegian law, and the specific patient consent, 2. data sharing with the specific data requester must be approved by the regional ethical committee (REC) in Norway, 3. the Data Protection Impact Assessment may require revision and 4. there must be a signed data transfer agreement between the institution of the data requester and NTNU. Depending on the intended use of the data, the data requester can also be required to establish a collaboration agreement with NTNU prior to data sharing. The source data underlying main and Supplementary Figs. are provided in Supplementary Data 5.

## Code availability

Original code was developed for pre-processing of the spatial transcriptomics data and are available from 10.5281/zenodo.13912230^59^. Other data analysis procedures, including software tools and software packages are described in “Methods”.
