# Supplementary material for: Spatial transcriptomics reveals strong association between SFRP4 and extracellular matrix remodeling in prostate cancer
Source: Commun Biol. 2024 Nov 8;7:1462. doi: 10.1038/s42003-024-07161-x (PMC11543834; doi:10.1038/s42003-024-07161-x)
Supplement: Supplementary file 1 — Supplementary Information [file 42003_2024_7161_MOESM1_ESM.pdf]

Supplementary to:

Spatial transcriptomics reveals strong association between *SFRP4* and extracellular matrix remodeling in prostate cancer

Maria K. Andersen[1,2]✉, Sebastian Krossa[1,3], Elise Midtbust[1,2], Christine A. Pedersen[1], Maximilian Wess[1], Therese S. Høiem[1], Trond Viset[4], Øystein Størkersen[4], Ingunn Nervik[5], Elise Sandsmark[1,6], Helena Bertilsson[3,7], Guro F. Giskeødegård[8], Morten B. Rye[2,5,9,10] and May-Britt Tessem[1,2]✉

1. Department of Circulation and Medical Imaging, NTNU - Norwegian University of Science and Technology, Trondheim, Norway
2. Clinic of Surgery, St. Olavs Hospital, Trondheim University Hospital, Trondheim, Norway
3. Central staff, St. Olavs Hospital HF, 7006, Trondheim, Norway
4. Department of Pathology, St. Olavs Hospital, Trondheim University Hospital, Trondheim, Norway
5. Department of Clinical and Molecular Medicine, NTNU - Norwegian University of Science and Technology, Trondheim, Norway
6. Department of Radiology and Nuclear Medicine, St. Olavs Hospital, Trondheim University Hospital, Trondheim, Norway
7. Central Norway Regional Health Authority, Stjørdal, Norway.
8. HUNT Center for Molecular and Clinical Epidemiology, Department of Public Health and Nursing, NTNU - Norwegian University of Science and Technology, Trondheim, Norway
9. Clinic of Laboratory Medicine, St.Olavs Hospital, Trondheim University Hospital, Trondheim, Norway
10. BioCore - Bioinformatics Core Facility, NTNU – Norwegian University of Science and Technology, Trondheim, Norway

✉ Corresponding authors: Maria K. Andersen (maria.k.andersen@ntnu.no), May-Britt Tessem (may-britt.tessem@ntnu.no)

Supplementary Methods

Proteomics on laser micro dissected (LMD) regions

Fresh frozen sections to be analyzed with MS proteomics were placed on sterile membrane slides (MMI, Eching, Germany). The sections were submerged in cold 70 % ethanol, dried for 30 s at room temperature, dehydrated in cold 100 % ethanol, air dried and stored at -80 °C.

The tissue sections were rinsed with ultrapure water (15 s) and stained with hematoxylin (30 s), followed by ultrapure water (15 s) and dehydration by sequentially washing with 50 %, 70 %, 95 % (15 s each), and 100 % ethanol (60 s). Directly after staining, the membrane glass slides were digitally scanned, vacuum packed and stored in -80 °C until LMD.

LMD were performed using a Leica LMD 6000 system (Wetzlar, Germany). Prior to cutting, the membrane slides were defrosted and air dried. LMD areas were cut and collected in 20 µl TE buffer within 2.5 hours after drying, centrifuged, and subsequently placed in -80 °C storage. A total of 114 areas were cut, including (but not limited to) 28 stroma regions, 30 normal gland regions and 37 cancer glands regions. Area of regions cut was in the range 39349 - 4165184 µm^2, while number of cells varied from 187 to 9171.

The LMD tissue areas were thawed, resuspended in 20 µl of lysis buffer (10 mM Tris-HCl pH 7.4, 1 mM EDTA, 0.02 % Zwittergent 3-16), heated for 60 min at 98 °C, and sonicated for 30 min. Iodoacetamide

(IAM; 15 mM, 15 min in the dark at room temperature) was used to alkylate cysteines after reduction of disulfide bridges (by dithiothreitol (DTT, 10 mM, 56 °C for 30 min)). The proteins were cleaved into peptides by overnight tryptic digestion (0.03 µg, 37 °C). Tryptic activity was terminated by acetic acid (HAC; final concentration of 0.5 %) and desalted with in-house packed C18 Stagetips (Empore, 3M). For LC-MS we used a TimsTOF Pro2 instrument coupled in-line with a nanoElute ultraperformance liquid chromatography (UPLC) system (Bruker Daltonics). Samples were analyzed in a randomized order, and approximately every tenth run an aliquoted prostate tissue standard (created from 5 different sections) were measured. Liquid chromatography was performed using a Pepsep C18 column (25 cm×150 µm, 1.5 µm; Bruker Daltonics) with a gradient of 0-37 % acetonitrile, 0.1 % formic acid for 75 min. The timsTof instrument was operated in the DDA PASEF mode with 10 PASEF scans per acquisition cycle and accumulation and ramp times of 100 ms each. The 'target value' was set to 20,000 and dynamic exclusion was activated and set to 0.4 min. The quadrupole isolation width was set to 2 Th for $m/z < 700$ and 3 Th for $m/z > 800$.

Proteins were identified using MaxQuant (version 2.0.3.1). Trypsin was chosen as proteolytic enzyme (max two missing cleavages). Carbamidomethylation of cysteine residues was set as fixed modifications and oxidation of methionine, acetylation of protein N-terminus, and deamidation of asparagine/glutamine was chosen as dynamic post-translational modifications. Each run in MaxQuant utilized *m/z* and retention time (RT)-values and aligned them to each of the samples (window: one minute match-between-run function and 20 min overall sliding window) using a clustering-based technique. The queries (including protein isoforms) were searched against the Human proteome database from Uniprot (downloaded June 2022; https://www.uniprot.org/proteomes/UP000005640). Contaminants were searched against MaxQuant's internal contaminants database using inbuilt Andromeda. Maximum false discovery rate was set to 1 % (FDR <0.01) for both peptides and proteins, where unique peptides with high confidence were used for final protein identification.

Supplementary Tables

Supplementary Table 1: Clinical data of study patients. Time is given as months. One patient lacked information on T-stage. Abbreviations: GG = grade group and PSA = prostate specific antigen.

ID	Status	Time to relapse	Follow up time w. PSA	Total follow up time	T-stage	Age at surgery	Average preoperative PSA	Operative GG
P01	Relapse	32	78	78	T3a	62	7.3	4
P02	Relapse	0	102	102	T2c	68	9	3
P03	Relapse	0	62	62	T3b	58	47.9	5
P04	Relapse-free	135	12	135	T2c	63	10	2
P05	Relapse-free	-	9	122	T2c	66	5.4	2
P06	Relapse-free	-	15	136	-	56	9.6	2
P07	Relapse	37	107	107	T3b	59	12.8	2
P08	Relapse	0	78	78	T3a	57	11	5
P09	Relapse	0	62	62	T3b	51	26.4	5
P10	Relapse-free	-	14	123	T2c	59	16.4	2
P11	Relapse	14	79	79	T3b	57	18.4	3
P12	Relapse	114	141	141	T2c	67	9.8	3
P13	Relapse	11	56	56	T2c	65	19	2
P14	Relapse	0	2	2	T3b	67	6.2	5
P15	Relapse	1	33	33	T3b	59	60	4
P16	Relapse-free	-	32	138	T2c	66	6.4	2
P17	Relapse	19	70	70	T3a	63	9.5	2
P18	Relapse	44	50	50	T2c	63	5.9	2
P19	Relapse	0	93	93	T2c	65	4.6	2
P20	Relapse	15	102	102	T2c	54	13.9	2
P21	Relapse	0	39	39	T3b	66	11.4	5
P22	Relapse	0	34	34	T3a	53	32.5	4
P23	Relapse	45	53	53	T3a	68	11.6	3
P24	Relapse	25	66	66	T3a	65	4.8	3
P25	Relapse	2	48	48	T3b	69	15.1	4
P26	Relapse	107	125	125	T2c	60	7.7	3
P27	Relapse	0	44	44	T3b	73	16.3	5
P28	Relapse-free	-	6	119	T2c	63	22.3	3
P29	Relapse-free	-	11	133	T2c	55	10.2	2
P30	Relapse	0	22	22	T2c	53	45.9	3
P31	Relapse	45	45	45	T3b	55	52.5	2
P32	Relapse-free	-	20	122	T2c	59	11.5	2
P33	Relapse	7	44	44	T3b	73	7.3	3
P34	Relapse	38	83	83	T2c	66	8.4	3
P35	Relapse-free	-	11	128	T2c	56	5.6	2
P36	Relapse	0	30	30	T3b	70	29	3
P37	Relapse-free	-	2	140	T2c	51	10.2	3

Supplementary Table 2: Differential analysis of *SFRP4* gene expression using linear mixed models (LMM) with patient origin as random effect. LMM was adjusted for stroma content and patient age. A logFC over 0 indicates increased expression in the more aggressive group compared.

Comparison	logFC	Mean expression	t	P-value	B
Cancer vs non-cancer samples	1.59	5.34	8.132	7.44E-14	20.888
Relapse vs relapse-free samples	0.87	5.34	1.904	0.058	-4.251
HG vs LG Cancer samples	0.33	5.84	1.14	0.256	-4.815
Relapse cancer vs relapse-free cancer samples	1.13	5.84	2.386	0.019	-3.377

Supplementary Table 3: Spearman correlation between methylation level and gene expression for *SFRP4*.

			Study cohort			TCGA cohort			ICGC cohort		
SiteID	Region	Position	ρ	P	Adj p	ρ	P	Adj p	ρ	P	Adj p
cg05682561	Promotor	37957021	-0.333	7.10E-03	0.047	-0.211	8.90E-07	8.01E-06	-0.255	1.83E-04	6.23E-04
cg06161814	Promotor	37956963	-0.366	0.003	0.030	-0.273	1.49E-10	2.68E-09	-0.337	5.57E-07	9.47E-06
cg04651042	Promotor	37956940	-0.316	0.011	0.044	-0.180	3.04E-05	1.37E-04			
cg09594069	Promotor	37956906	-0.264	0.035	0.078	-0.175	4.83E-05	1.74E-04	-0.286	2.54E-05	2.16E-04
cg21122375	Promotor	37956869	0.190	0.132	0.203	-0.111	0.010	0.021	-0.178	9.77E-03	0.028
cg01689311	Promotor	37956850	-0.219	0.082	0.136	-0.111	0.010	0.023	-0.270	7.55E-05	4.28E-04
cg07669460	Promotor	37956688	-0.272	0.030	0.074	-	-	-	-	-	-
cg22826141	Promotor	37956648	-0.100	0.431	0.575	-0.009	0.829	0.829	-0.084	0.228	0.352
cg10806140	Promotor	37956554	-0.290	0.020	0.067	0.030	0.487	0.516	-0.077	0.264	0.374
cg25783719	Promotor	37956434	-0.253	0.044	0.088	0.078	0.071	0.091	-0.051	0.461	0.560
cg08261094	Promotor	37956276	-0.525	0.000	0.000	-0.082	0.058	0.087	0.027	0.698	0.742
cg20019546	Promotor	37955824	0.289	0.021	0.059	0.056	0.193	0.218	0.154	0.025	0.061
cg14846368	Gene body	37955623	0.018	0.888	0.935	0.103	0.017	0.028	-0.103	0.135	0.255
cg19166347	Gene body	37955598	0.053	0.675	0.794	0.116	7.60E-03	0.020	0.031	0.657	0.744
cg23569180	Gene body	37955508	-0.156	0.218	0.311	-0.125	3.81E-03	0.011	-0.106	0.125	0.266
cg20191905	Gene body	37952539	-0.012	0.925	0.925						
cg23169784	Gene body	37947061	0.248	0.048	0.088	-0.111	0.011	0.019	-0.022	0.757	0.757
cg13400306	Gene body	37947051	0.019	0.882	0.980	-0.080	0.065	0.090	-0.096	0.164	0.278
Average promoter	Promotor	-	-0.328	8.20E-03	0.041	-0.198	4.27E-06	2.56E-05	-0.258	1.56E-04	6.62E-04
Average gene body	Gene body	-	0.094	0.460	0.575	0.070	0.109	0.131	-0.063	0.363	0.475

Supplementary Table 4: Differential analysis of *SFRP4* gene methylation using linear mixed models (LMM) with patient origin as random effect. LMM was adjusted for stroma content and patient age. Higher and lower methylation levels in the most aggressive group compared is indicated.

	Cancer vs non-cancer samples			Relapse vs relapse-free samples			High-grade vs low-grade Cancer samples			Relapse cancer vs relapse-free cancer samples		
SiteID	P	Adj_P	Higher or lower	P	Adj P	Higher or lower	P	Adj P	Higher or Lower	P	Adj P	Higher or Lower
cg05682561	2.55E-03	7.35E-03	LOWER	0.856	0.997	HIGHER	0.704	0.939	LOWER	0.583	0.827	LOWER
cg06161814	8.54E-05	1.29E-03	LOWER	0.895	0.997	LOWER	0.196	0.552	LOWER	0.406	0.827	LOWER
cg04651042	7.16E-04	4.78E-03	LOWER	0.916	0.997	LOWER	0.280	0.622	LOWER	0.518	0.827	LOWER
cg09594069	1.87E-03	7.35E-03	LOWER	0.376	0.982	LOWER	0.163	0.542	LOWER	0.112	0.827	LOWER
cg21122375	0.196	0.301	LOWER	0.987	0.997	LOWER	0.144	0.542	LOWER	0.709	0.828	LOWER
cg01689311	0.018	0.046	LOWER	0.688	0.982	LOWER	0.616	0.939	LOWER	0.241	0.827	LOWER
cg07669460	0.002	0.007	LOWER	0.575	0.982	LOWER	0.944	0.957	LOWER	0.295	0.827	LOWER
cg22826141	0.928	0.928	HIGHER	0.461	0.982	HIGHER	0.957	0.957	HIGHER	0.489	0.827	HIGHER
cg10806140	0.039	0.086	LOWER	0.619	0.982	LOWER	0.644	0.939	LOWER	0.188	0.827	LOWER
cg25783719	0.607	0.714	LOWER	0.584	0.982	HIGHER	0.679	0.939	HIGHER	0.746	0.828	HIGHER
cg08261094	2.57E-03	7.35E-03	LOWER	0.555	0.982	LOWER	0.472	0.858	HIGHER	0.662	0.827	LOWER
cg20019546	0.438	0.548	HIGHER	0.125	0.646	HIGHER	0.002	0.047	HIGHER	0.654	0.827	HIGHER
cg14846368	0.124	0.248	HIGHER	0.032	0.630	HIGHER	0.221	0.552	HIGHER	0.165	0.827	HIGHER
cg19166347	0.740	0.779	HIGHER	0.210	0.840	HIGHER	0.059	0.335	HIGHER	0.351	0.827	HIGHER
cg23569180	0.137	0.249	LOWER	0.868	0.997	LOWER	0.067	0.335	HIGHER	0.556	0.827	LOWER
cg20191905	0.219	0.308	HIGHER	0.327	0.982	LOWER	0.363	0.727	LOWER	0.826	0.852	LOWER
cg23169784	0.176	0.293	HIGHER	0.063	0.631	HIGHER	0.934	0.957	HIGHER	0.055	0.827	HIGHER
cg13400306	0.647	0.719	LOWER	0.672	0.982	LOWER	0.839	0.957	LOWER	0.852	0.852	HIGHER
Average promoter	1.29E-04	1.29E-03	LOWER	0.997	0.997	HIGHER	0.923	0.957	LOWER	0.429	0.827	LOWER
Average gene body	0.231	0.308	HIGHER	0.129	0.646	HIGHER	0.040	0.335	HIGHER	0.343	0.827	HIGHER

Supplementary Table 5: Spearman correlation analysis between average *SFRP4* gene expression from stroma spots pr sample and average Wnt target genes in epithelial spots from the same sample.

Wnt pathway gene target	Spearman coefficient	P-value	Adjusted p
MYC	-0.277	0.125	0.500
CCND1	0.549	0.001	0.011
LBH	0.023	0.902	0.953
MMP7	-0.011	0.953	0.953
CD44	0.128	0.485	0.776
ID2	0.085	0.642	0.856
JAG1	-0.193	0.289	0.578
SOX9	0.226	0.212	0.566

Supplementary Table 6: Estimation of fibrosis in stroma after Masson's trichrome staining. Row and position indicate sample placement in Supplementary Figure 18. Stroma area is the area of the stroma annotation in Qupath. A threshold for pink stain for the most optimal segmentation was selected for each sample. Area pink stain in stroma is the area of segmented pink stain within the stroma annotation. Fraction pink is given by area of pink stain divided by stroma area, while the fraction of blue is given as 1 minus fraction of pink stain.

sample_id	Row	Position	Stroma area	Threshold	Area pink stain in stroma	Fraction pink	Fraction blue
P07_1	1	a	1646836	0.2	777389.4	0.4721	0.5279
P08_8	1	b	2810889.8	0.25	1283133.5	0.4565	0.5435
P04_6	1	c	4215701.1	0.3	1906960.3	0.4523	0.5477
P06_7	1	d	3035393.3	0.2	2152290.6	0.7091	0.2909
P04_3	2	a	3526248.7	0.2	1726240.9	0.4895	0.5105
P06_1	2	b	1572818.1	0.25	809732.1	0.5148	0.4852
P07_6*	2	c	1211278.2	0.15	681651.3	0.5628	0.4372
P08_6	2	d	2205258.8	0.3	656304	0.2976	0.7024
P07_5	3	a	877091.7	0.15	489408.6	0.5580	0.4420
P08_3	3	b	2141848.9	0.15	1464287.4	0.6837	0.3163
P04_4	3	c	1581506.8	0.2	907324.4	0.5737	0.4263
P06_8	3	d	2739580.4	0.15	2069698.2	0.7555	0.2445
P04_5	4	a	2211001.7	0.15	1721154.3	0.7785	0.2215
P06_6	4	b	3262593.8	0.15	1843832.1	0.5651	0.4349
P07_7	4	c	3398042.7	0.17	2105357.8	0.6196	0.3804
P08_4	4	d	2239426.3	0.2	758813.4	0.3388	0.6612
P22_1	5	a	963854.7	0.2	448228.9	0.4650	0.5350
P30_1	5	b	1355370.8	0.15	666669.8	0.4919	0.5081
P28_3	5	c	2613827.4	0.2	1450870.7	0.5551	0.4449
P27_1	5	d	4206694	0.2	2203027.3	0.5237	0.4763
P27_2	6	a	1605120.6	0.3	1003929.8	0.6255	0.3745
P28_4	6	b	1337230	0.15	746039.9	0.5579	0.4421
P30_3	6	c	2092603.3	0.3	1286783.6	0.6149	0.3851
P22_2	6	d	1464837.2	0.25	647456.2	0.4420	0.5580
P28_2	7	a	2686016.1	0.2	1150781.7	0.4284	0.5716
P27_3	7	b	3426774.7	0.2	532449.2	0.1554	0.8446
P22_3	7	c	4765282.2	0.15	2678017.4	0.5620	0.4380
P30_4	7	d	3423642.4	0.2	2132851	0.6230	0.3770
P30_2	8	a	1954416.7	0.15	1493232.4	0.7640	0.2360
P22_4	8	b	4212012	0.25	1640022.9	0.3894	0.6106
P27_4	8	c	1765846.1	0.3	875841	0.4960	0.5040
P28_1	8	d	1088180.8	0.3	284644.9	0.2616	0.7384

* Excluded from analysis due to poor quality

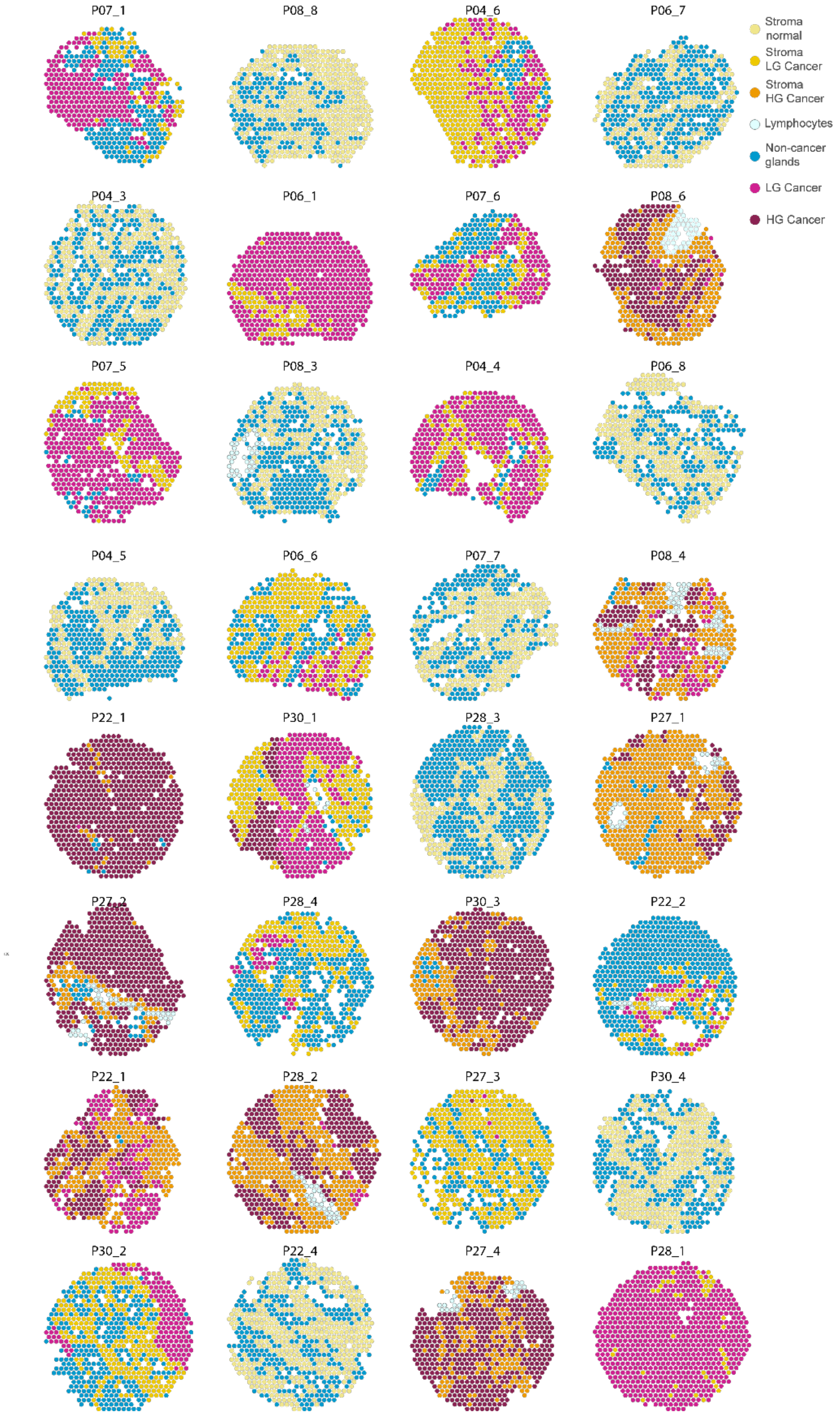

Supplementary Figure 1: Histopathology classifications for each spatial transcriptomics spot. Low grade (LG) cancer included Gleason grade group 1 and 2, while high grade (HG) cancer represents grade groups 3 through 5. Stroma spots were assigned according to if they coappeared in samples with normal glands, LG or HG cancer glands.

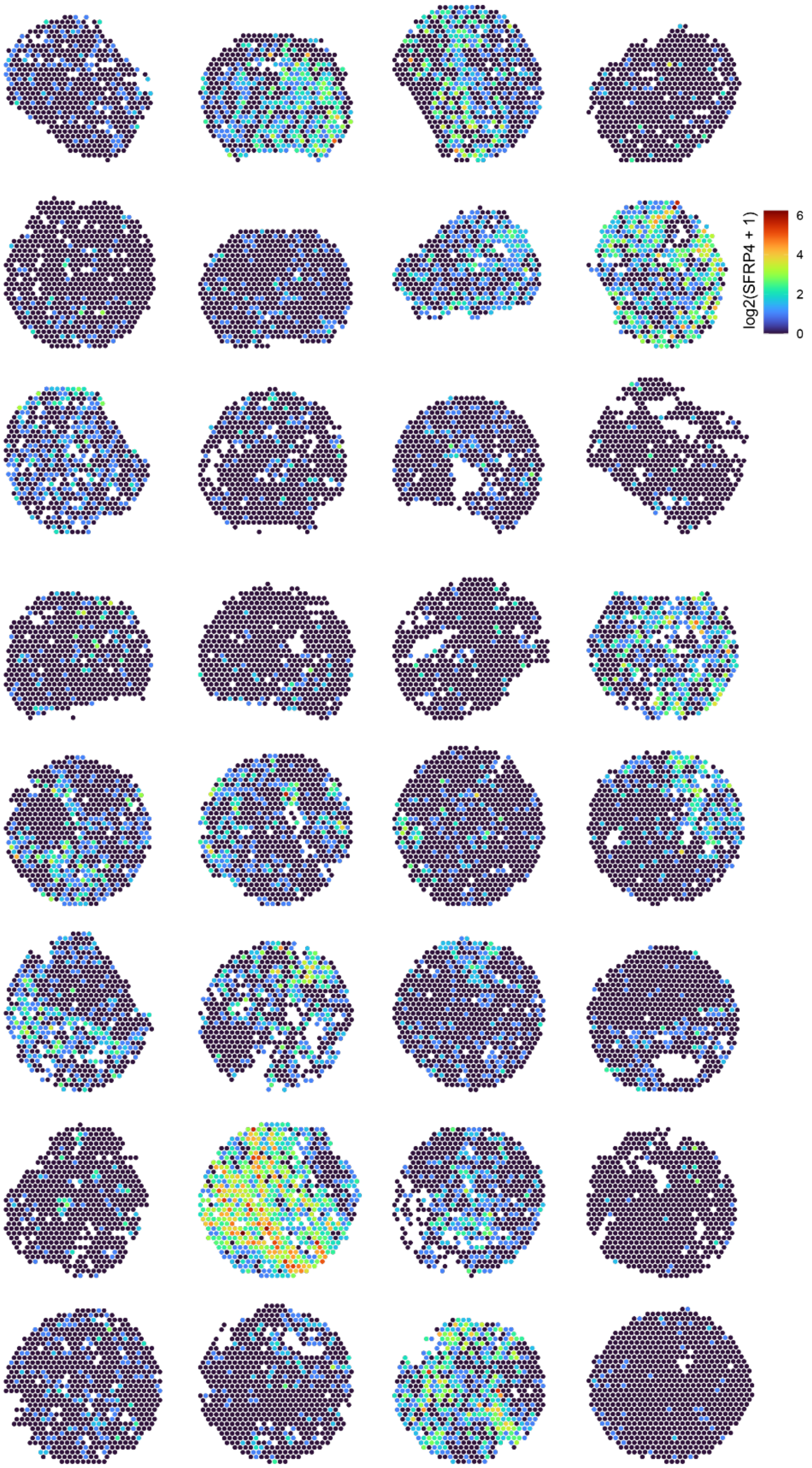

Supplementary Figure 2: Spatial gene expression distribution of *SFRP4*. Gene counts are cell count normalized and $\log_2$-transformed.

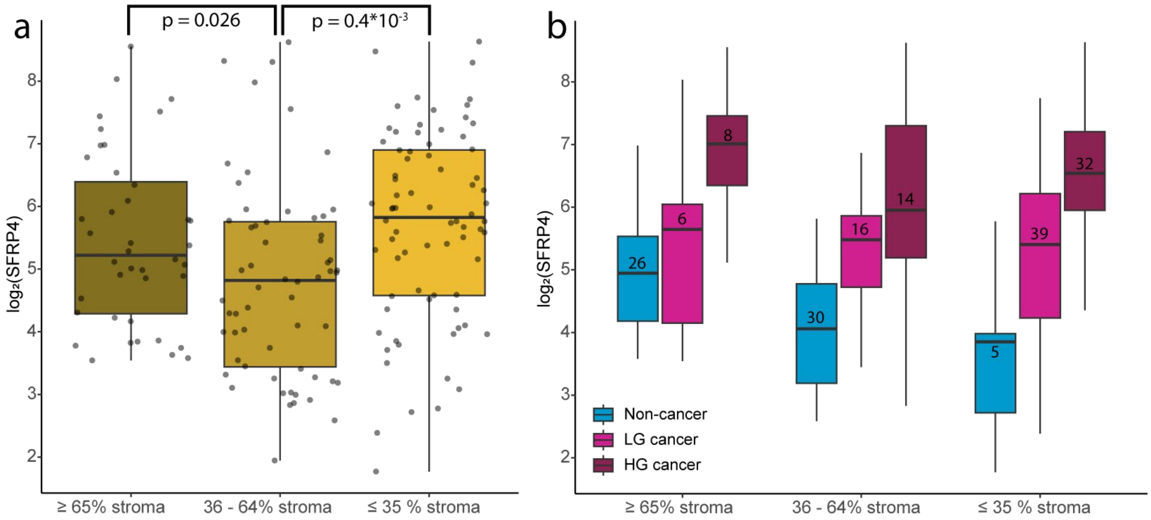

Supplementary Figure 3: Gene expression of *SFRP4* in bulk samples with different stroma content. boxplots showing counts per million normalized *SFRP4* levels a) independent of sample type and b) separated on sample type. In a), the p-value was calculated with a Wilcoxon rank sum-test. In b), low grade (LG) cancer samples included Gleason grade group 1 and 2, while high grade (HG) cancer represents grade groups 3 through 5. Number of samples are given for each boxplot in b).

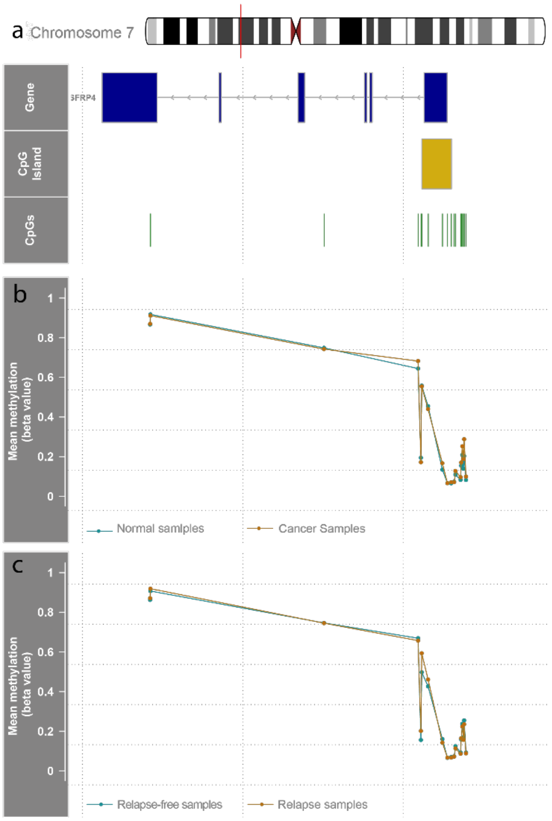

Supplementary Figure 4: *SFRP4* DNA Methylation. a) Diagram shows chromosome and genome location of the *SFRP4* gene. Mean methylation values are presented for b) comparing normal to cancer samples and c) comparing control to relapse samples.

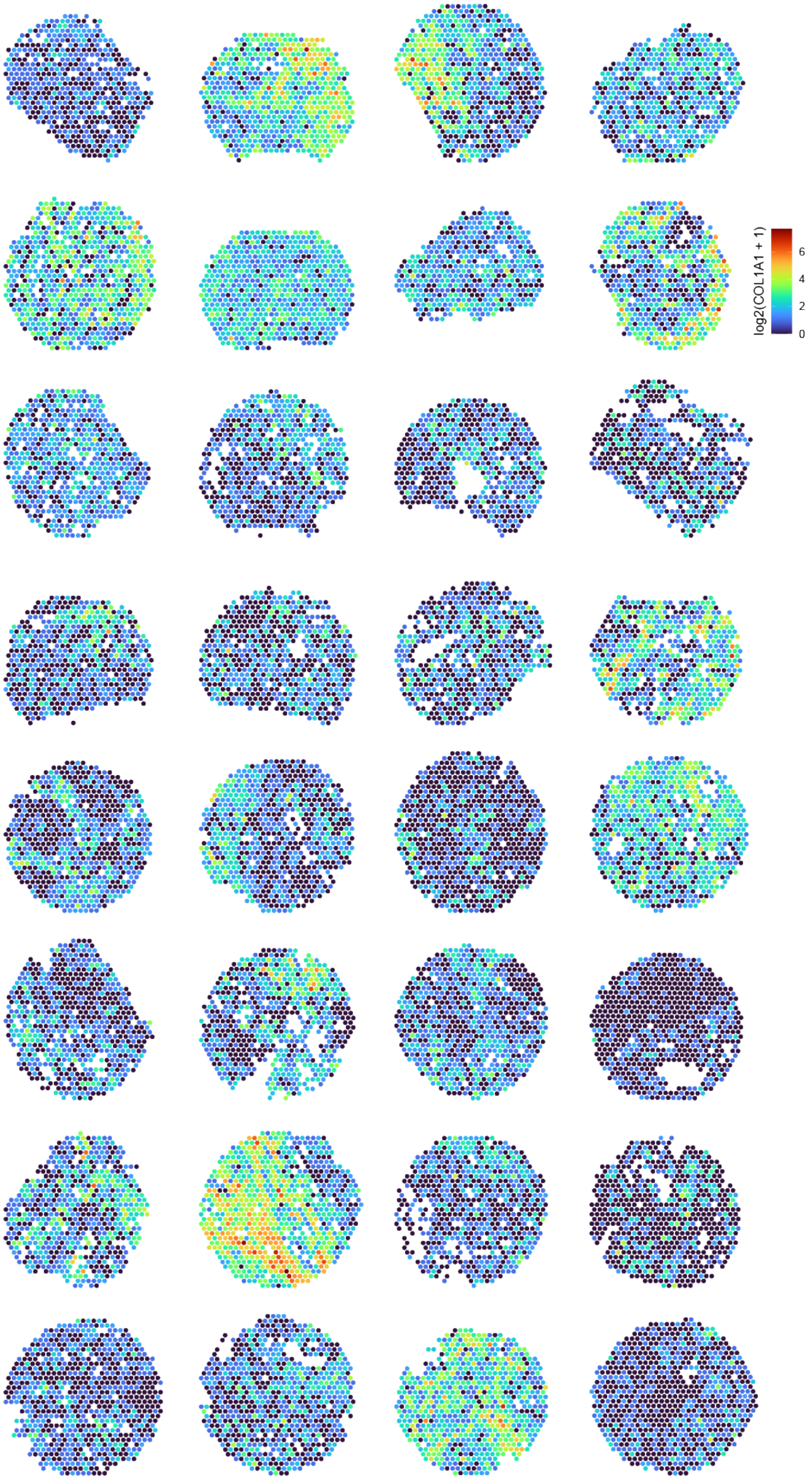

Supplementary Figure 5: Spatial gene expression distribution of *COL1A1*. Gene counts are cell count normalized and log_2-transformed.

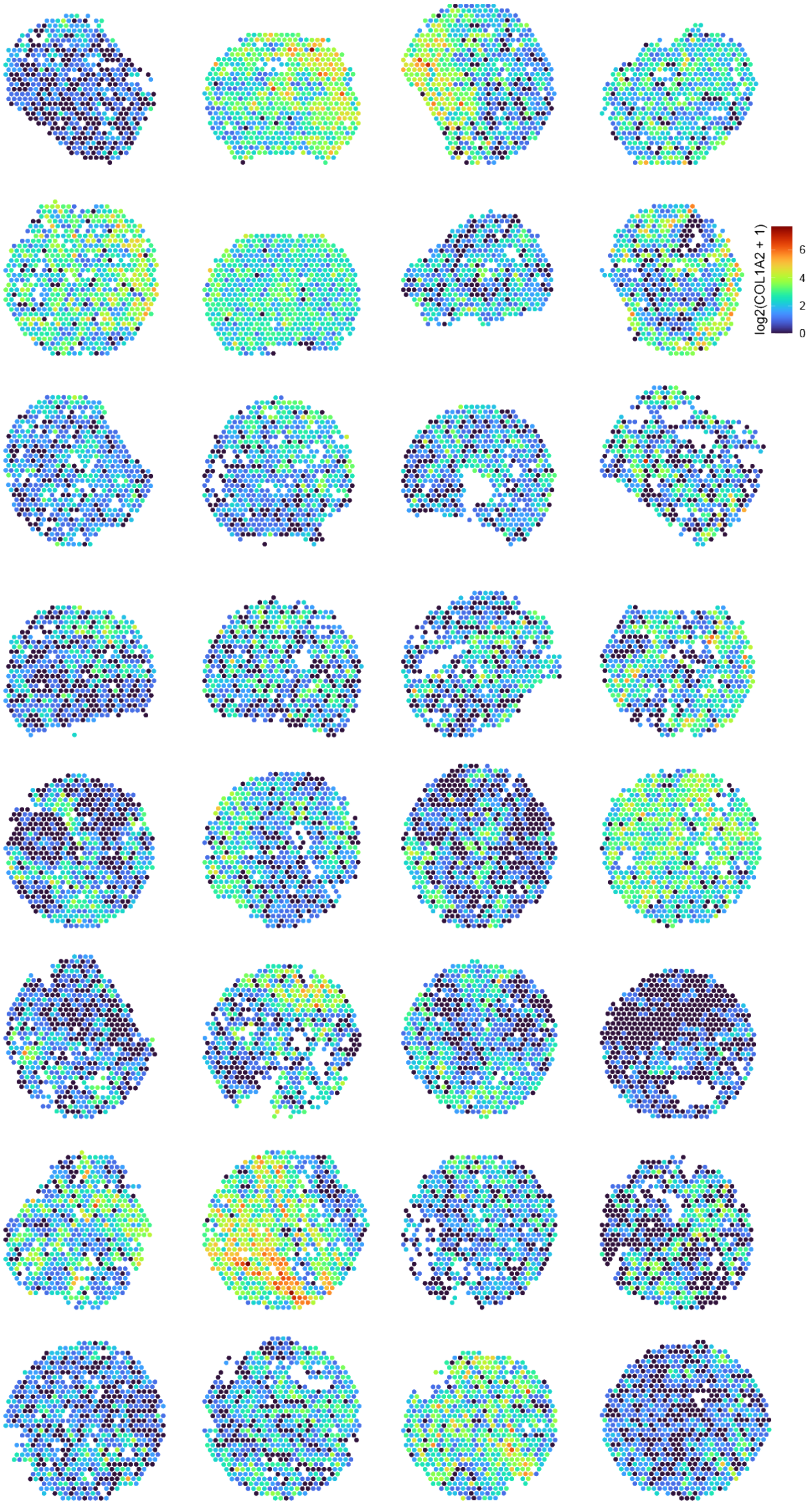

Supplementary Figure 6: Spatial gene expression distribution of *COL1A2*. Gene counts are cell count normalized and log_2-transformed.

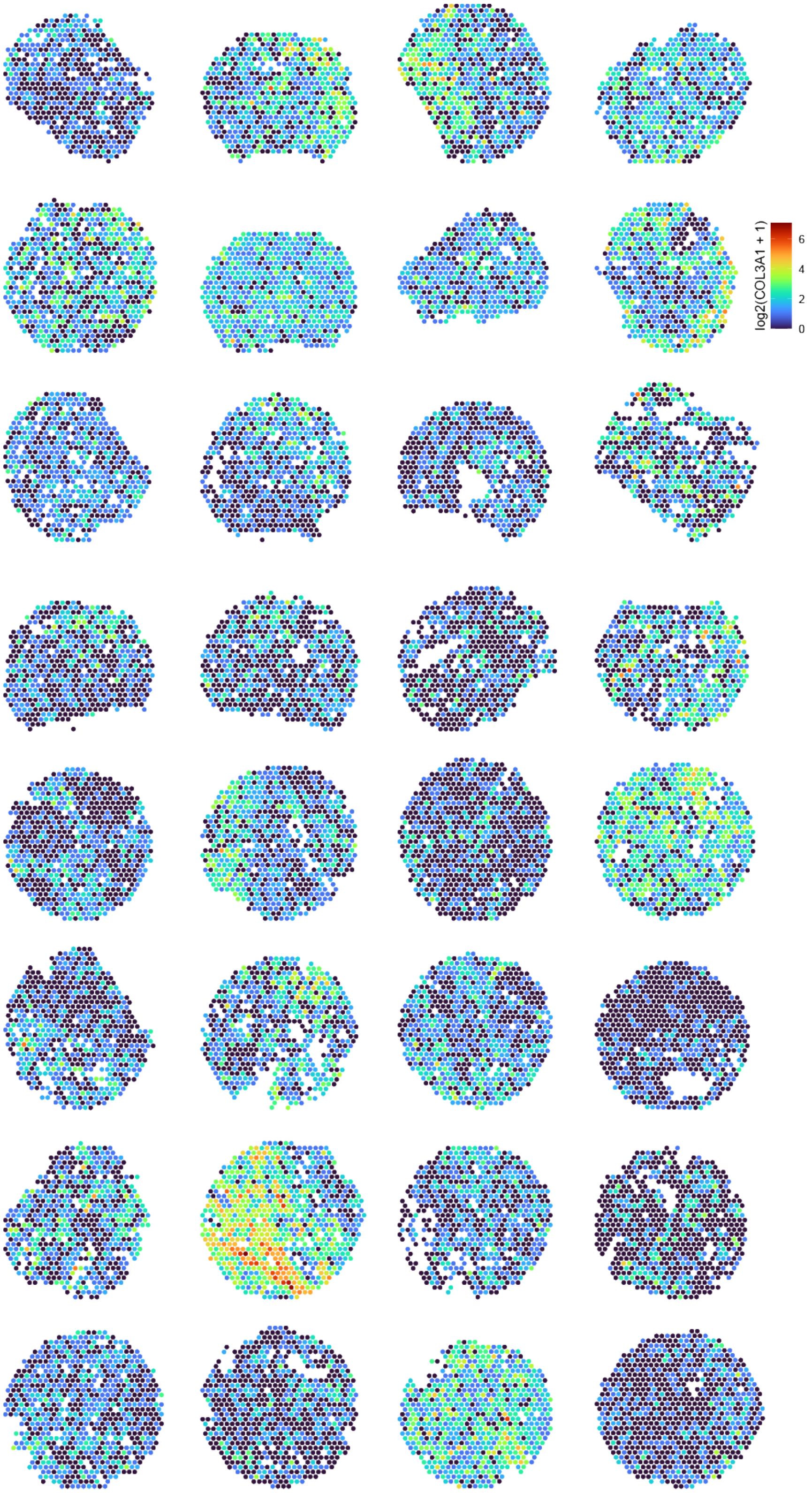

Supplementary Figure 7: Spatial gene expression distribution of *COL3A1*. Gene counts are cell count normalized and log_2-transformed.

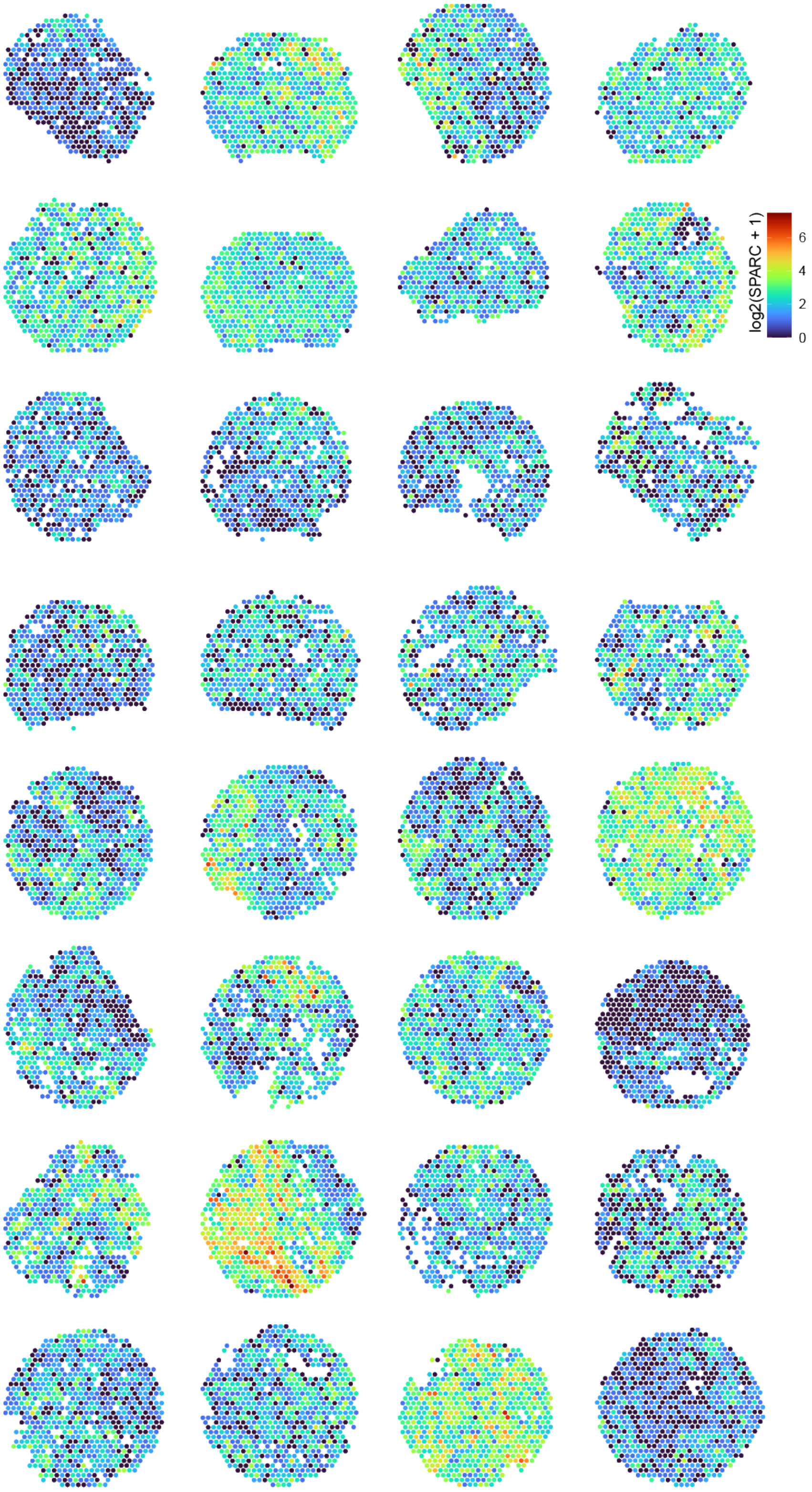

Supplementary Figure 8: Spatial gene expression distribution of *SPARC*. Gene counts are cell count normalized and $\log_2$-transformed.

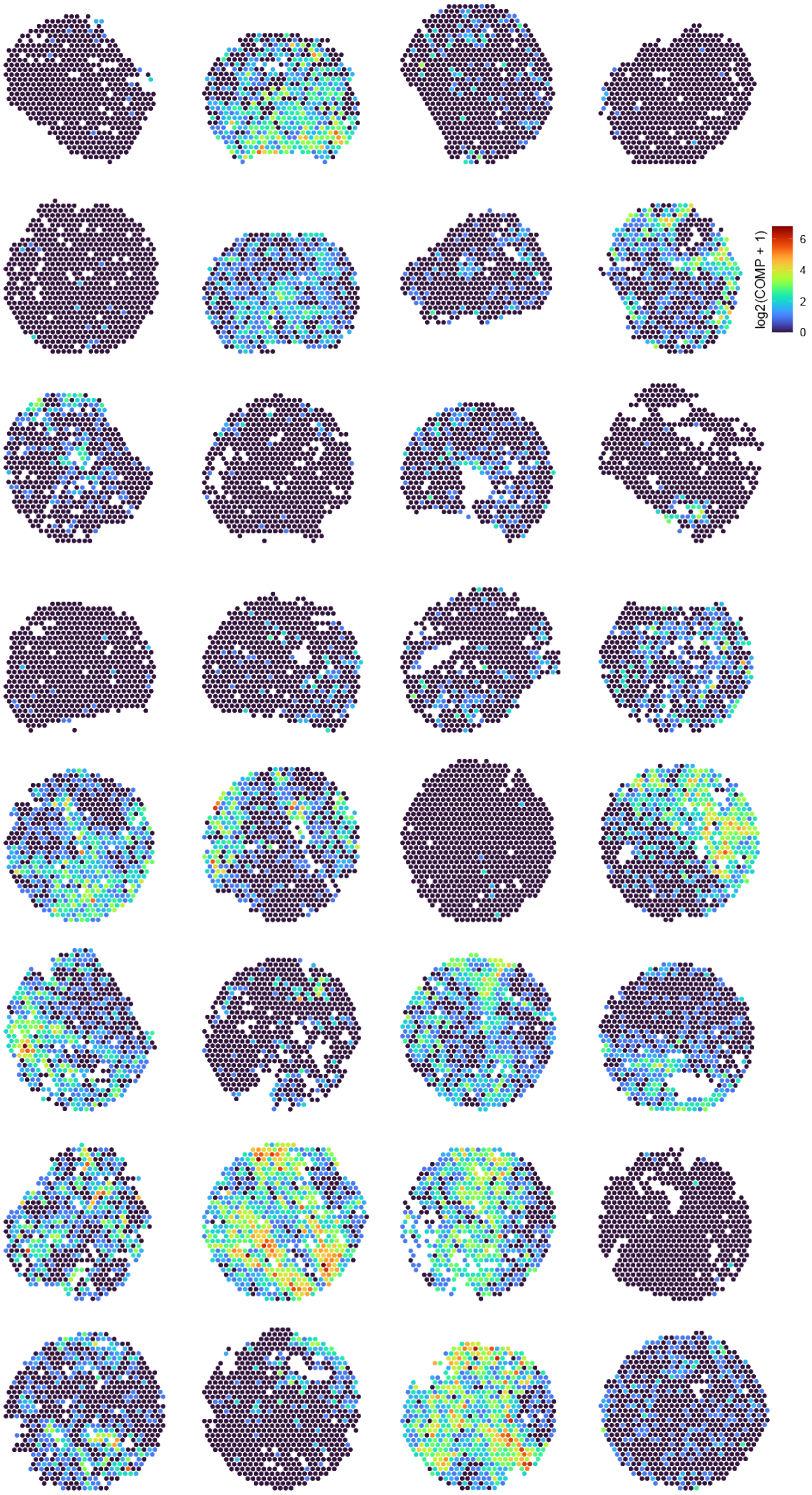

Supplementary Figure 9: Spatial gene expression distribution of *COMP*. Gene counts are cell count normalized and log_2-transformed.

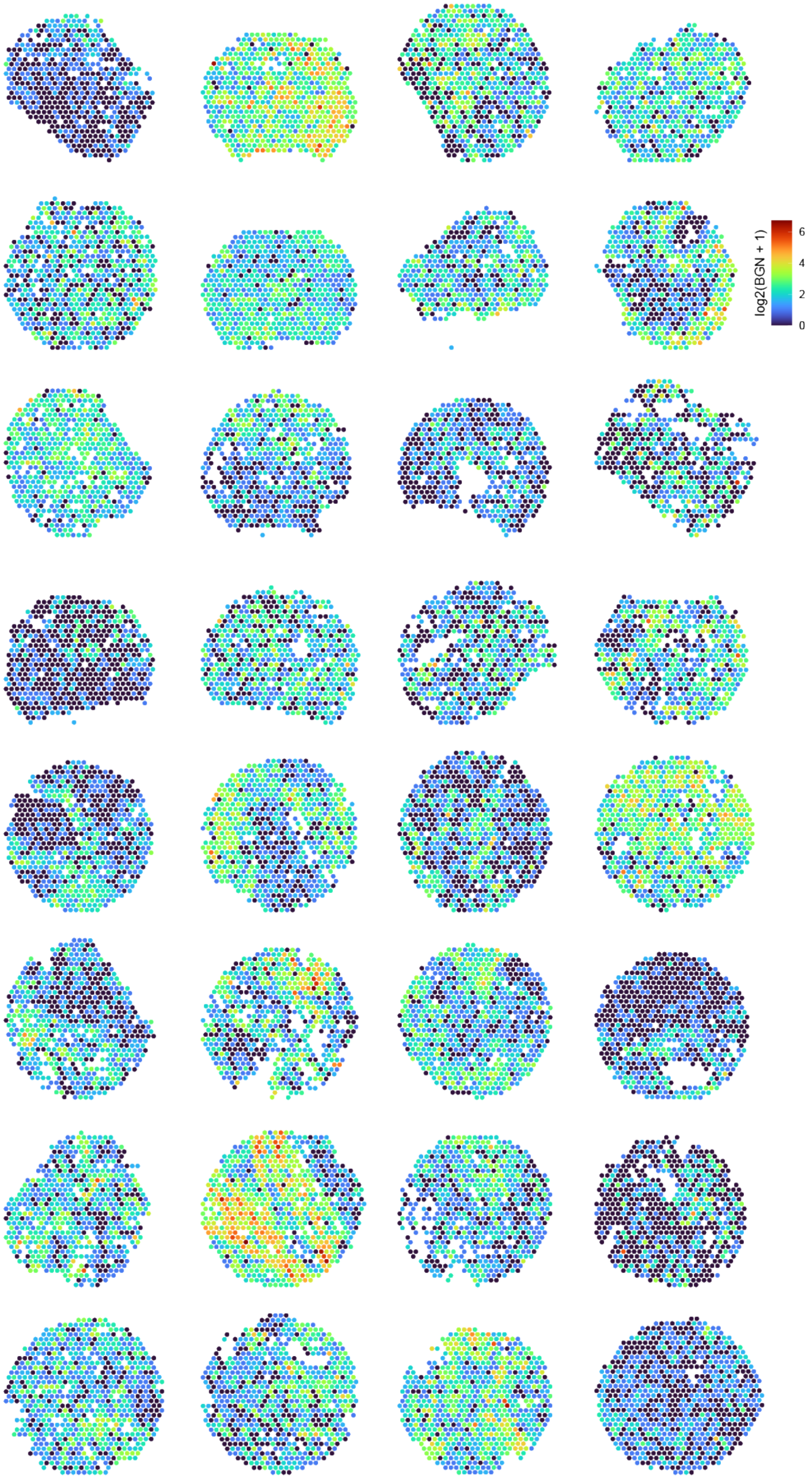

Supplementary Figure 10: Spatial gene expression distribution of *BGN*. Gene counts are cell count normalized and log_2-transformed.

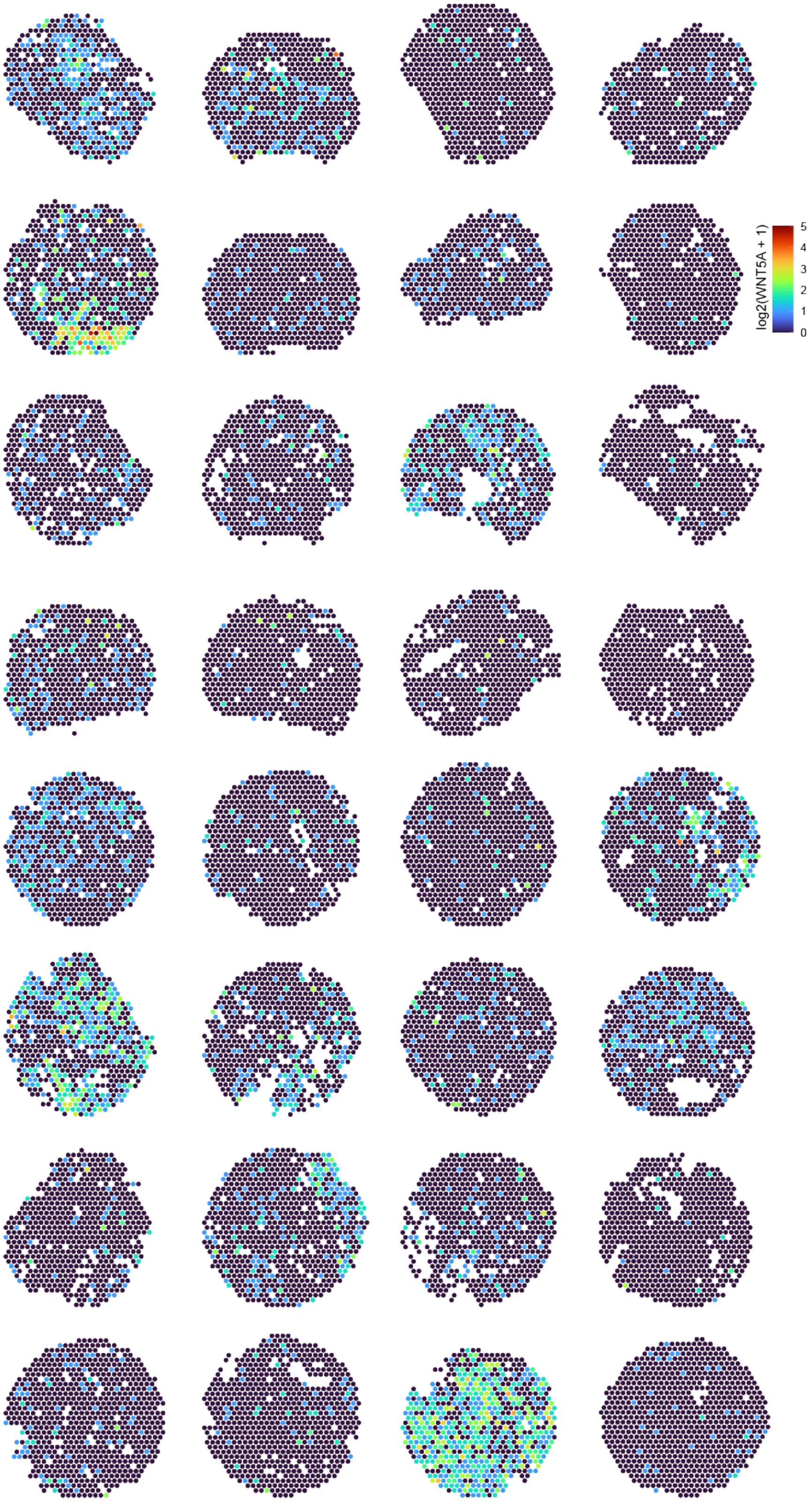

Supplementary Figure 11: Spatial gene expression distribution of *WNT5A*. Gene counts are cell count normalized and $\log_2$-transformed.

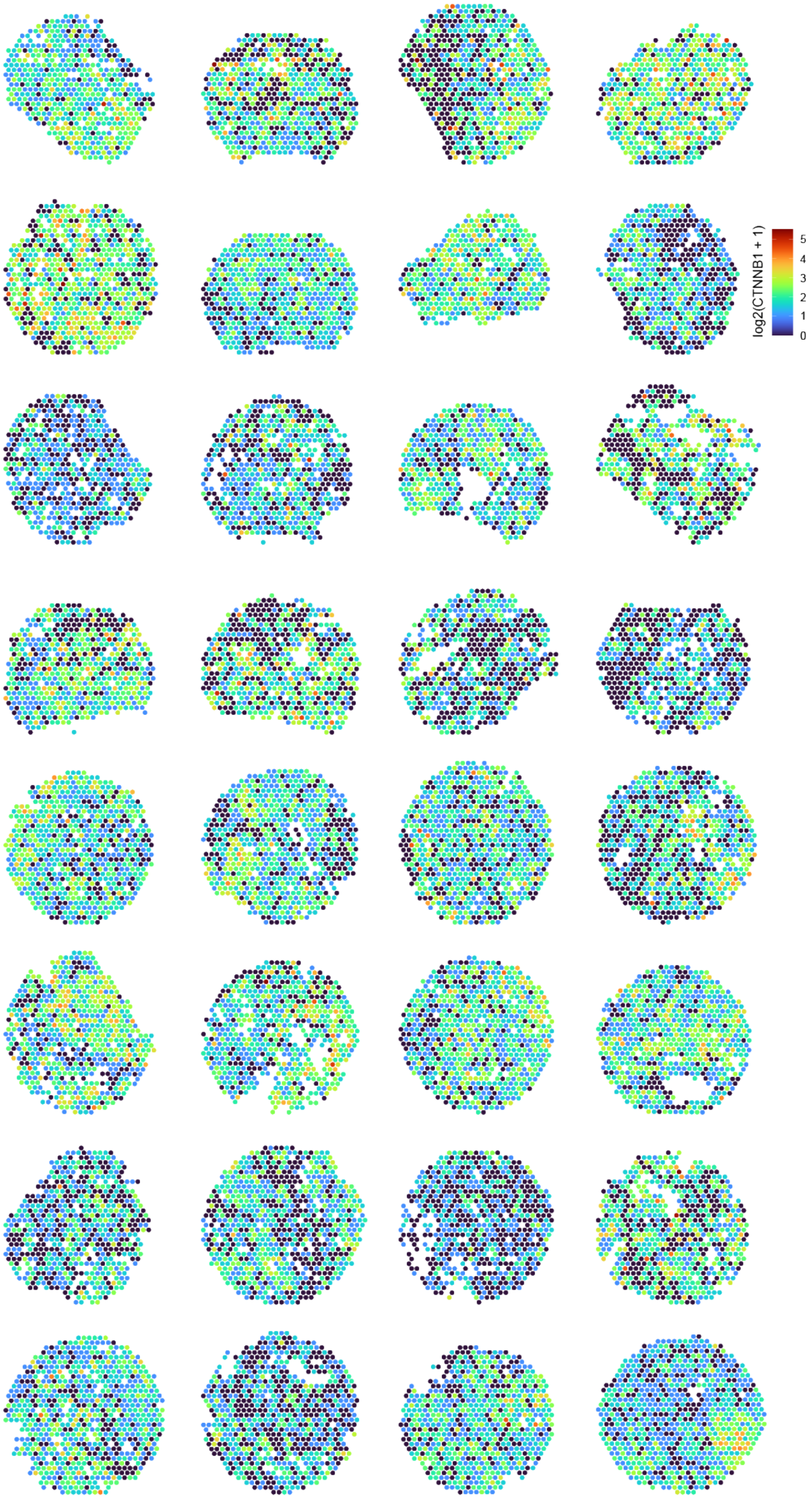

Supplementary Figure 12: Spatial gene expression distribution of *CNNB1*. Gene counts are cell count normalized and log_2-transformed.

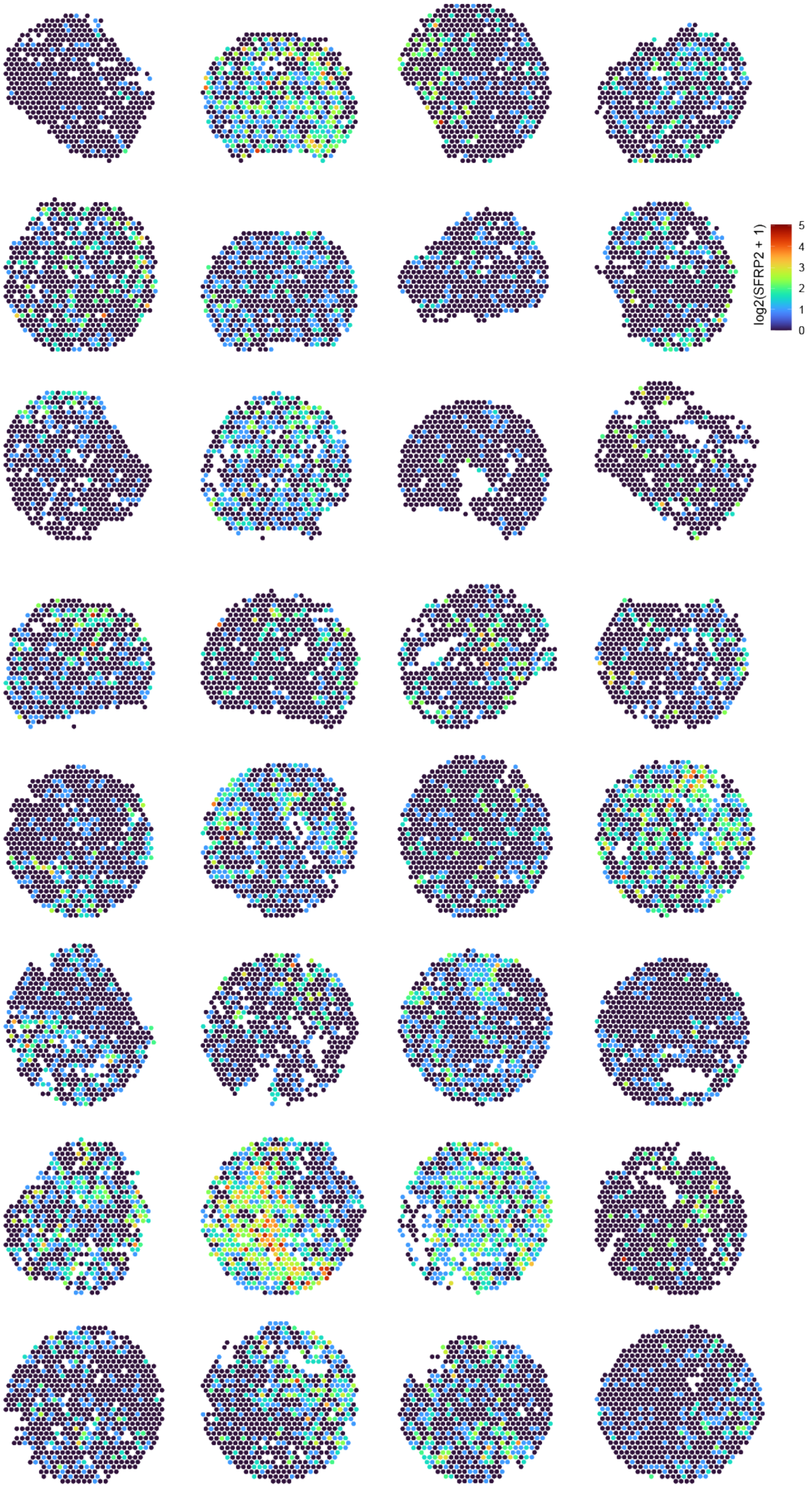

Supplementary Figure 13: Spatial gene expression distribution of *SFRP2*. Gene counts are cell count normalized and log_2-transformed.

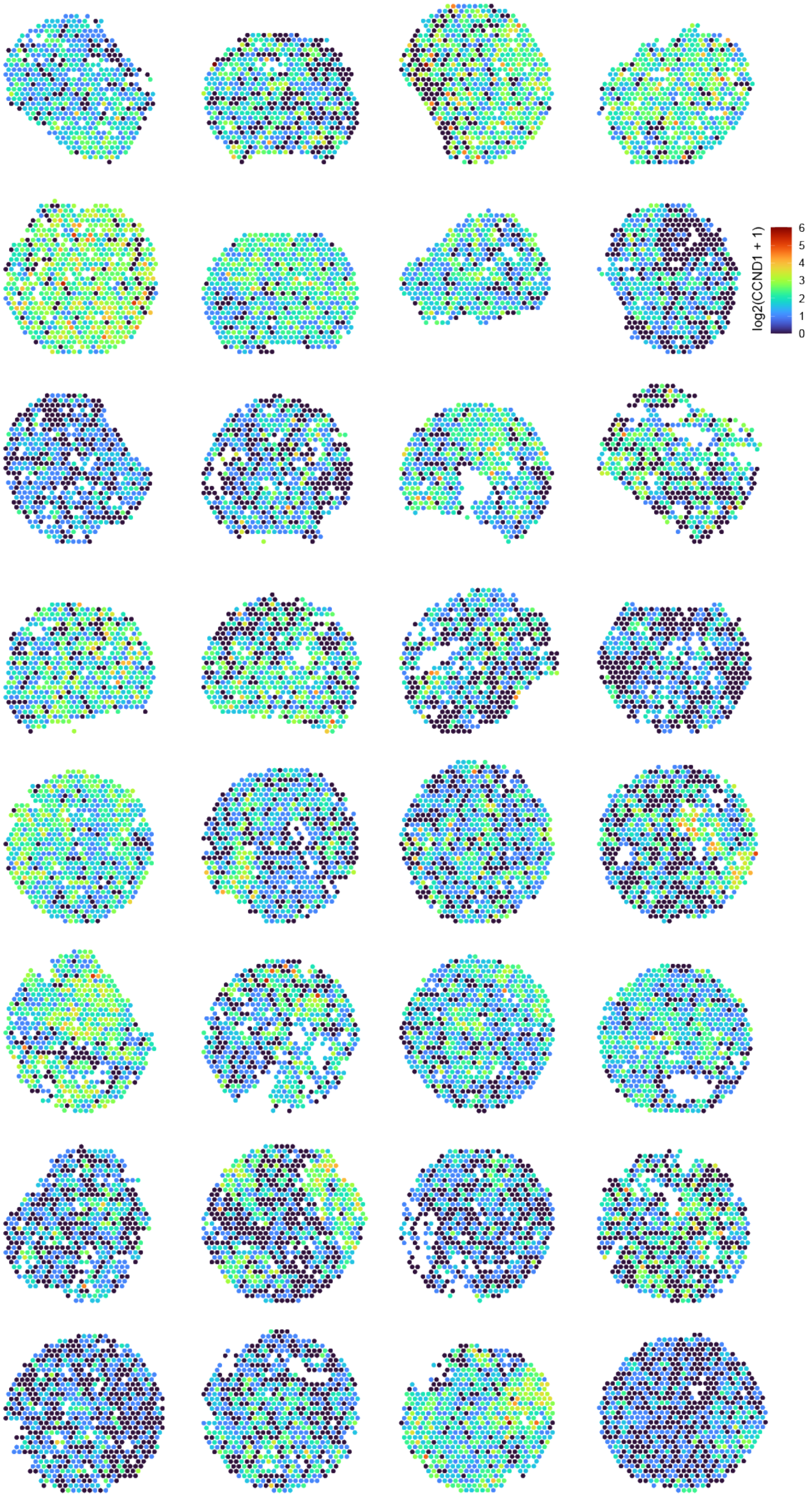

Supplementary Figure 14: Spatial gene expression distribution of *CCND1*. Gene counts are cell count normalized and $\log_2$-transformed.

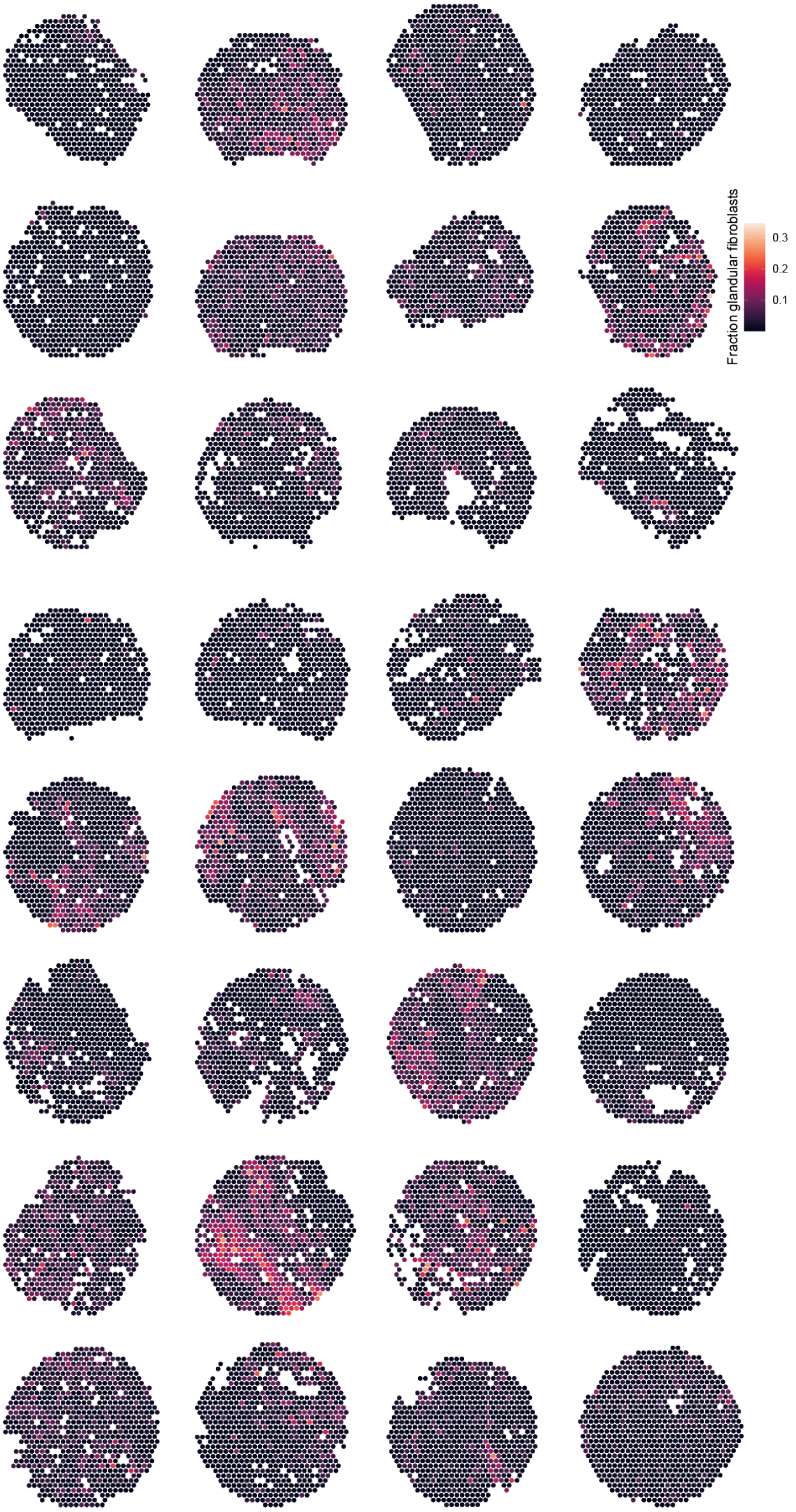

Supplementary Figure 15: Estimated cell fraction of glandular fibroblasts in each spatial transcriptomics spot.

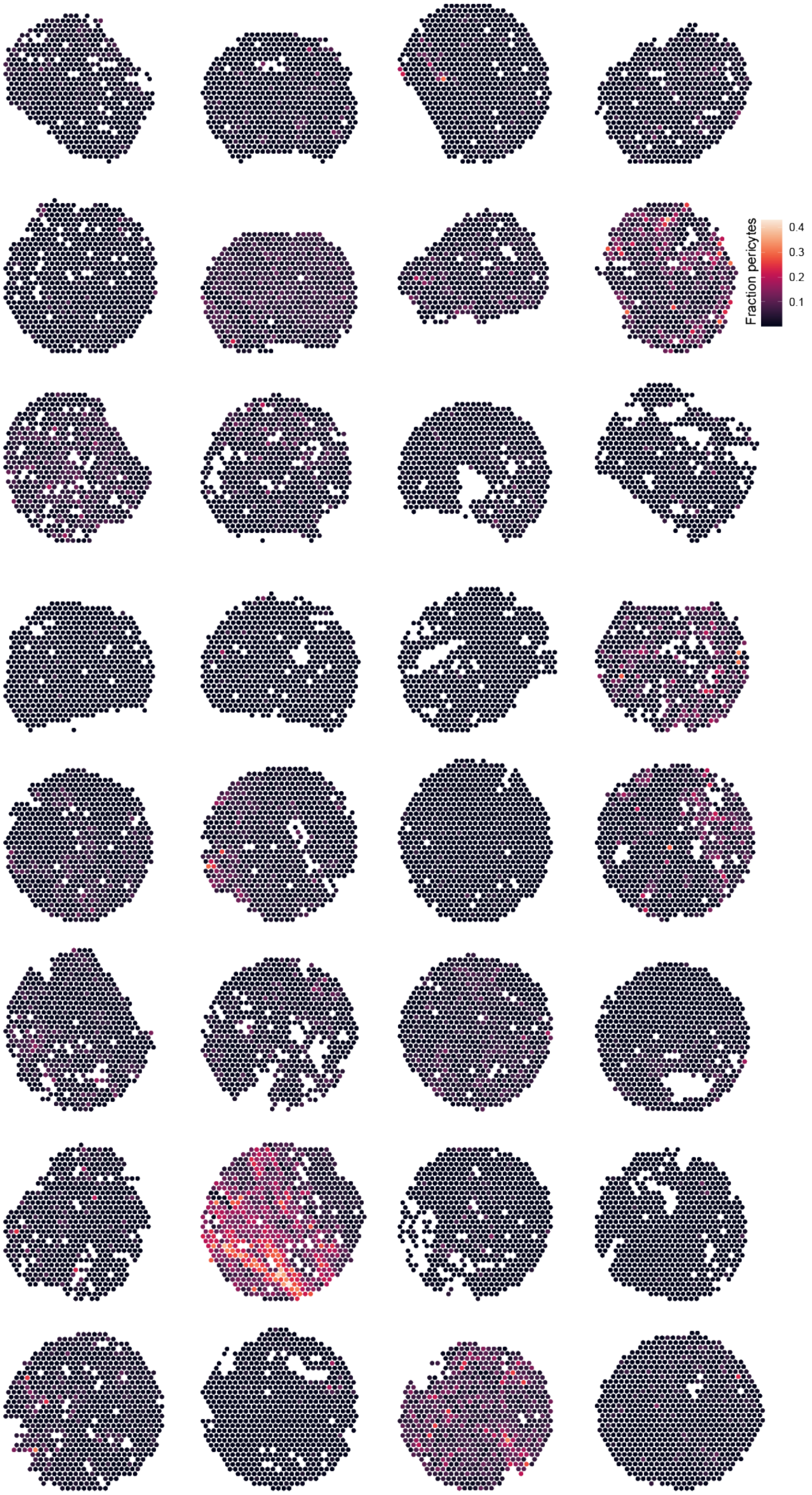

Supplementary Figure 16: Estimated cell fraction of pericyte in each spatial transcriptomics spot.

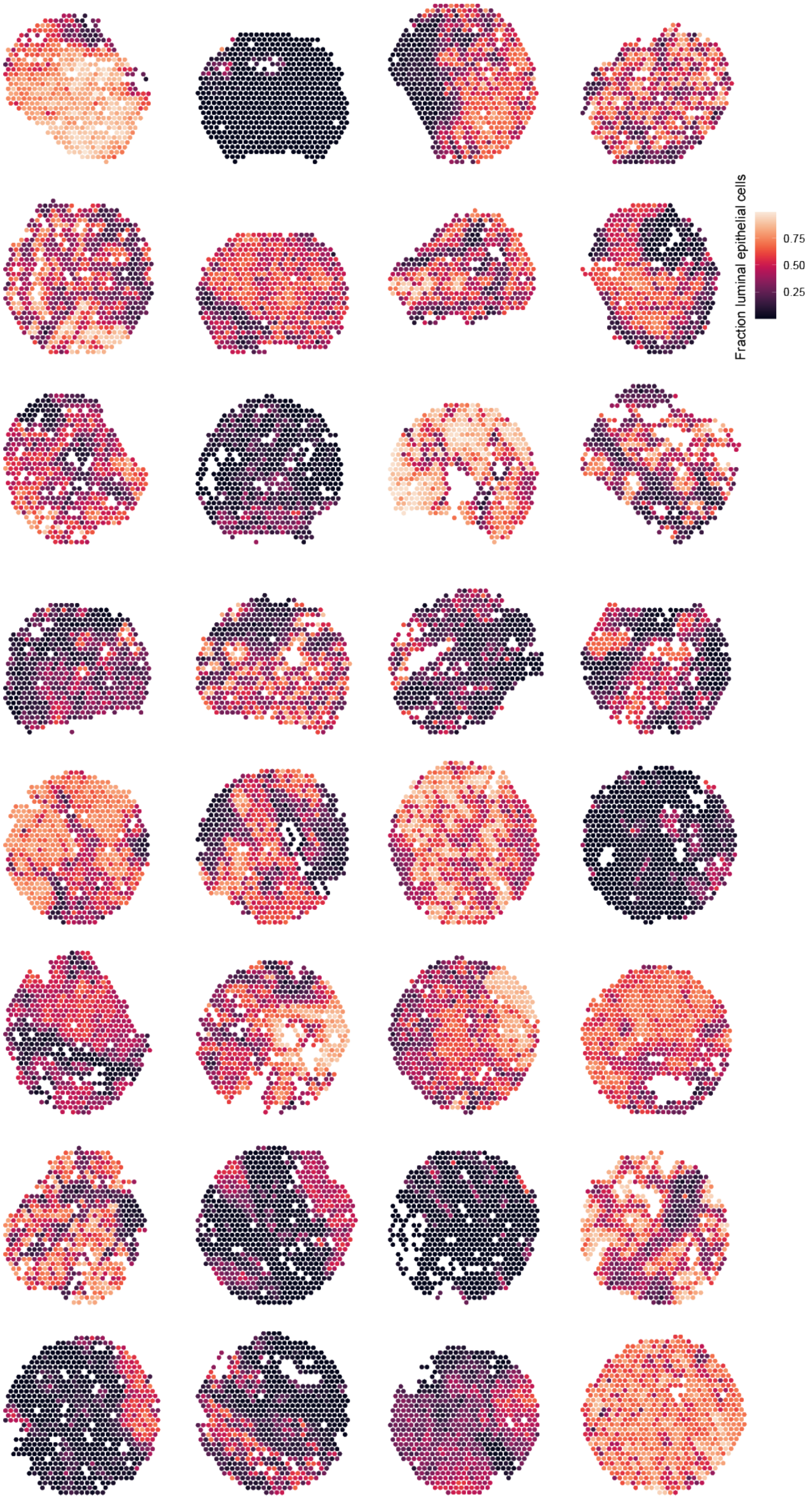

Supplementary Figure 17: Estimated cell fraction of luminal epithelial cells in each spatial transcriptomics spot.

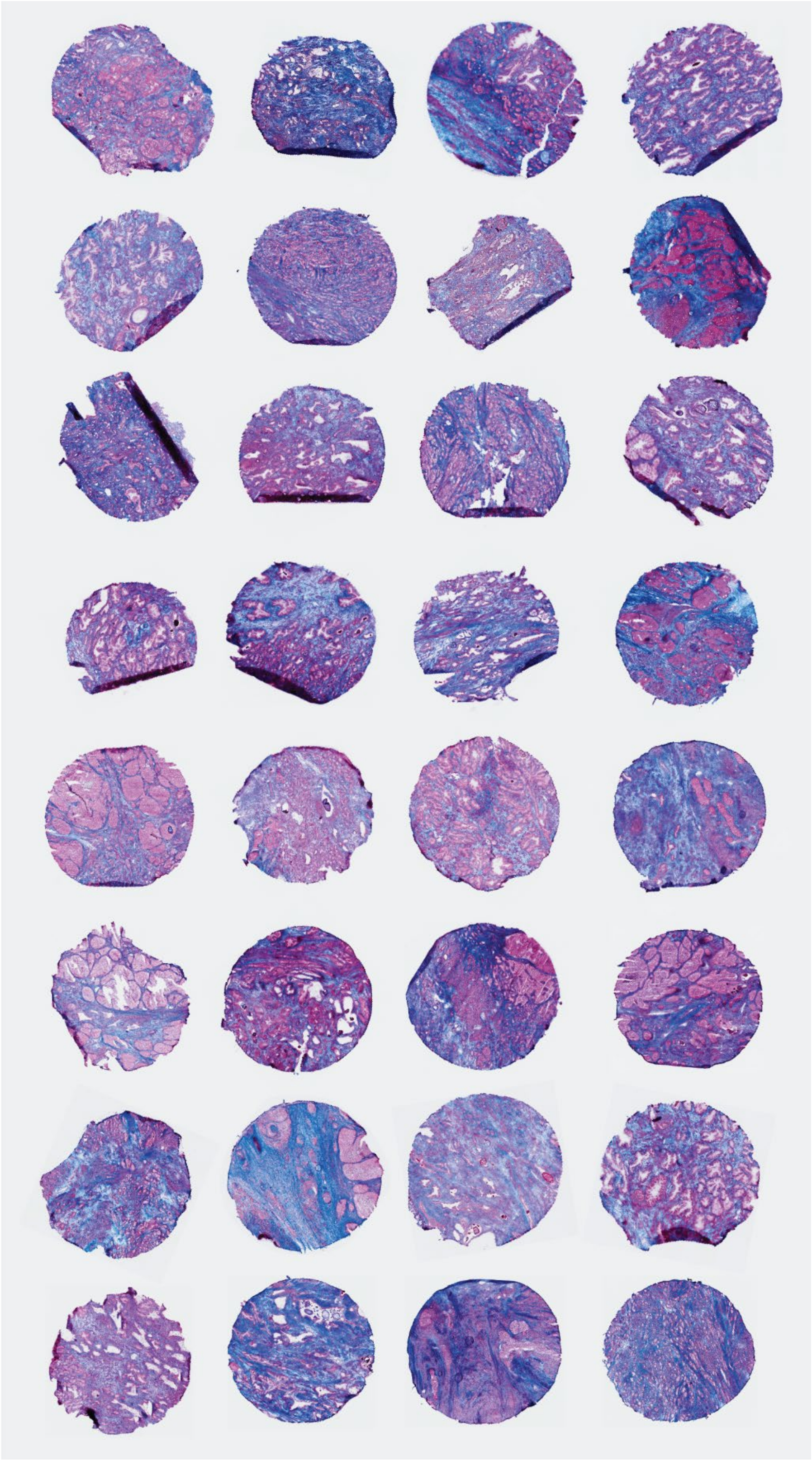

Supplementary Figure 18: Masson's trichrome stain. Staining was performed on serial sections of the same samples used for spatial transcriptomics.
