# Supplementary material for: Spatial transcriptomics reveals strong association between SFRP4 and extracellular matrix remodeling in prostate cancer
Source: Commun Biol. 2024 Nov 8;7:1462. doi: 10.1038/s42003-024-07161-x (PMC11543834; doi:10.1038/s42003-024-07161-x)
Supplement: Supplementary file 2 — Description of Additional Supplementary Files [file 42003_2024_7161_MOESM2_ESM.pdf]

Description of Additional Supplementary Files

File name: Supplementary Data 1

Description: Sample and multi-omics analysis overview.

File name: Supplementary Data 2

Description: Differential analysis of stroma spots from the spatial transcriptomics (ST) dataset.

File name: Supplementary Data 3

Description: List of detected proteins in MS-based LMD proteomics data.

File name: Supplementary Data 4

Description: Spearman correlation analysis. SFRP4 gene expression correlated with other genes in ST data, SFRP4 gene expression correlated with other genes in single cell transcriptomics data and SFRP4 gene expression correlated with estimated cell type fractions in ST data.

File name: Supplementary Data 5

Description: The source data behind the graphs in the paper.
