# Supplementary material for: Spatial transcriptomics reveals strong association between SFRP4 and extracellular matrix remodeling in prostate cancer
Source: Commun Biol. 2024 Nov 8;7:1462. doi: 10.1038/s42003-024-07161-x (PMC11543834; doi:10.1038/s42003-024-07161-x)
Supplement: Supplementary file 8 — Reporting summary [file 42003_2024_7161_MOESM8_ESM.pdf]

nature portfolio

Corresponding author(s): Maria K. Andersen

Last updated by author(s): Oct 25, 2024

Reporting Summary

Nature Portfolio wishes to improve the reproducibility of the work that we publish. This form provides structure for consistency and transparency in reporting. For further information on Nature Portfolio policies, see our Editorial Policies and the Editorial Policy Checklist.

Statistics

For all statistical analyses, confirm that the following items are present in the figure legend, table legend, main text, or Methods section.

n/a	Confirmed	
☐	☒	The exact sample size (*n*) for each experimental group/condition, given as a discrete number and unit of measurement
☐	☒	A statement on whether measurements were taken from distinct samples or whether the same sample was measured repeatedly
☐	☒	The statistical test(s) used AND whether they are one- or two-sided<br>*Only common tests should be described solely by name; describe more complex techniques in the Methods section.*
☐	☒	A description of all covariates tested
☐	☒	A description of any assumptions or corrections, such as tests of normality and adjustment for multiple comparisons
☐	☒	A full description of the statistical parameters including central tendency (e.g. means) or other basic estimates (e.g. regression coefficient) AND variation (e.g. standard deviation) or associated estimates of uncertainty (e.g. confidence intervals)
☐	☒	For null hypothesis testing, the test statistic (e.g. *F*, *t*, *r*) with confidence intervals, effect sizes, degrees of freedom and *P* value noted<br>*Give P values as exact values whenever suitable.*
☒	☐	For Bayesian analysis, information on the choice of priors and Markov chain Monte Carlo settings
☒	☐	For hierarchical and complex designs, identification of the appropriate level for tests and full reporting of outcomes
☐	☒	Estimates of effect sizes (e.g. Cohen's *d*, Pearson's *r*), indicating how they were calculated

Our web collection on statistics for biologists contains articles on many of the points above.

Software and code

Policy information about availability of computer code

Data collection	No software was used for data collection
Data analysis	Spatial transcriptomics data: 10x genomics space ranger software package (version 1.0.0) for converting BCL to FASTQ files, using the human reference transcriptome GRCh38 (version 3.0.0). Original code was developed for pre-processing of the spatial transcriptomics data to classify each spot into histopathology classes. These python-scripts are available from DOI: 10.5281/zenodo.13912230.<br>Bulk transcriptomics: FASTQ files were filtered and trimmed using fastp v0.20.0. For sequence alignment the STAR tool was used against a reference set (Ensembl, GRCh38 release 92). featureCounts was used to extract gene counts from sequence reads according to the same reference set.<br>Methylation data: minfi packge in R was used for normalization and IlluminaHumanMethylationEPICanno.ilm10b4.hg19 was used to find SFRP4 sites.<br>Histopathology annotation: QuPath v 0.2.3 for viewing and annotating digital stained scans of tissue sections. Each spatial transcriptomics spot were merged with the histology images and classified into its histology class using in-house developed python scripts available from: https://github.com/sekro/spatial_transcriptomics_toolbox<br>Differential analysis: Performed in R using the packages limma and edgeR.<br>Cell type fraction estimation: Stereoscope tool developed by Anderson et al (Communications Biology, 2020).<br>Survival analysis: Gene expression cut-off identified with the Cutoff Finder Tool (link) and Kaplan-Meier plot performed with the survival package in R.

For manuscripts utilizing custom algorithms or software that are central to the research but not yet described in published literature, software must be made available to editors and reviewers. We strongly encourage code deposition in a community repository (e.g. GitHub). See the Nature Portfolio guidelines for submitting code & software for further information.

Data

Policy information about availability of data

All manuscripts must include a data availability statement. This statement should provide the following information, where applicable:

- Accession codes, unique identifiers, or web links for publicly available datasets
- A description of any restrictions on data availability
- For clinical datasets or third party data, please ensure that the statement adheres to our policy

The data generated and analyzed in this study includes sensitive information, and its management must comply with the General Data Protection Regulation (GDPR), Norwegian law, and the specific patient consent and ethical approval. Consequently, the data is legally subjected to restricted access. Raw and processed transcriptomics and DNA methylation data have been deposited at Federated European Genome-Phenome Archive (FEGA) Norway and are findable on the EGA portal (ega-archive.org) under the study ID EGAS50000000413. The spatial transcriptomics, bulk transcriptomics and DNA methylation data are deposited as separate datasets with the accession numbers EGAD50000000603, EGAD50000000604 and EGAD50000000605, respectively. Data access can be requested through the EGA portal, where any data request will be processed through a data access committee at NTNU. The proteomics data is not externally archived as there are currently no suitable public data repository that accept sensitive proteomics data and that meets the data sharing criteria postulated by the study's ethical approval, patient consent, GDPR and Norwegian law. The proteomics data can be requested via email to maria.k.andersen@ntnu.no and may-britt.tessem@ntnu.no. For both archived and non-archived data, access will only be granted after the following steps have been achieved; 1. the data requester and the intended use of the data must comply with GDPR regulation, Norwegian law, and the specific patient consent, 2. data sharing with the specific data requester must be approved by the regional ethical committee (REC) in Norway, 3. the Data Protection Impact Assessment (DPIA) may require revision and 4. there must be a signed data transfer agreement between the institution of the data requester and NTNU. Depending on the intended use of the data, the data requester can also be required to establish a collaboration agreement with NTNU prior to data sharing. The source data underlying main and supplementary figures are provided in Supplementary Data 5.

Research involving human participants, their data, or biological material

Policy information about studies with human participants or human data. See also policy information about sex, gender (identity/presentation), and sexual orientation and race, ethnicity and racism.

Reporting on sex and gender	Prostate cancer only affects biological male individuals and all tissue donors in our study are therefore men
Reporting on race, ethnicity, or other socially relevant groupings	We have not reported race, ethnicity nor other social groupings for the tissue donors of this study, nor is any of this information available to us.
Population characteristics	The tissue donors were men diagnosed with prostate cancer aged 53-73 (median 63) years at the time for surgery. They did not receive an treatment prior to surgery and tissue donation
Recruitment	Men undergoing radical prostatectomy as a treatment for prostate cancer at St. Olav's Hospital are routinely asked by Bioabank1 if they are willing to donate a small part of the removed prostate (2mm cross-section) for research. The donation does not place any additional burden on the patients in terms of time or extra procedures, and we are not aware of any recruitment bias. Tissue samples were then retrospectively selected by us several years after surgery.
Ethics oversight	This research was approved by the regional ethical committee of Central Norway (identifier 2017/576), and all methods were performed according to national and EU ethical regulations.

Note that full information on the approval of the study protocol must also be provided in the manuscript.

Field-specific reporting

Please select the one below that is the best fit for your research. If you are not sure, read the appropriate sections before making your selection.

☒ Life sciences ☐ Behavioural & social sciences ☐ Ecological, evolutionary & environmental sciences

For a reference copy of the document with all sections, see nature.com/documents/nr-reporting-summary-flat.pdf

Life sciences study design

All studies must disclose on these points even when the disclosure is negative.

Sample size	The findings presented in this manuscript is one of the results from the ERC-funded project "Tissue is the issue" (grant agreement no. 758306). This project uses a highly comprehensive multi-omics approach to uncover the biological mechanisms at play in prostate cancer tissue. Most omics-methods come at a substantial cost and any multi-omics project need to balance sample size and number of included omics method. In this project we have ensured that our sample sizes for the different omics-methods are comparable or better than other omics publications which are using either spatial transcriptomics, epigenomics and bulk transcriptomics. Further, we have used several large publicly available datasets to validate that our findings are robust.<br><br>Data generated in the study:<br>Spatial transcriptomics, proteomics and Masson's Trichrome staining: 32 samples from 8 patients

	Bulk transcriptomics: 176 samples from 37 patients<br>Bulk Methylation: 64 samples from 16 patients<br><br>Publically available data<br>Single cell transcriptomics: 83 451 cells<br>Bulk transcriptomics TCGA: 532 samples<br>Bulk transcriptomics ICGC: 210 samples<br>Proteomics ICGC: 63 samples
Data exclusions	For the Masson's Trichrome stain analysis, one section was excluded due to poor tissue integrity, making quantification of staining intensities not possible. For the spatial transcriptomics data: Spots that were >50% outside the tissue border, had >50% tissue fold, >80% luminal space or >50% uncertain area were excluded. All spots with less than 1 percentile (41 genes) unique genes expressed and/or less than 2 percentile (116 reads) total reads were removed. Genes with less than 10 reads in less than 10 spots were filtered out.
Replication	No technical replication was performed in this study. Cancer tissue sections are heterogeneous and serial sections are therefore never identical. True technical replication of spatial experiments are therefore not possible.<br><br>For each statistical test the input sample was either a spatial transcriptomics spot (spatial data) or whole tissue sample (bulk data). Samples within the same group being tested against another group are considered as biological replicates to each other. For examples, when comparing cancer samples to normal samples, a cancer sample from patient 1 and a cancer sample from patient 2 were biological replicates
Randomization	This is a retrospective study where the clinical end-points were already known, and randomization of participants are therefore not relevant. We did randomize the order in which samples were analyzed to limit potential technical batch effects.
Blinding	Since this is a retrospective study, blinding was not necessary

Reporting for specific materials, systems and methods

We require information from authors about some types of materials, experimental systems and methods used in many studies. Here, indicate whether each material, system or method listed is relevant to your study. If you are not sure if a list item applies to your research, read the appropriate section before selecting a response.

Materials & experimental systems

n/a	Involved in the study
☒	☐ Antibodies
☒	☐ Eukaryotic cell lines
☒	☐ Palaeontology and archaeology
☒	☐ Animals and other organisms
☐	☒ Clinical data
☒	☐ Dual use research of concern
☒	☐ Plants

Methods

n/a	Involved in the study
☒	☐ ChIP-seq
☒	☐ Flow cytometry
☒	☐ MRI-based neuroimaging

Clinical data

Policy information about clinical studies

All manuscripts should comply with the ICMJE guidelines for publication of clinical research and a completed CONSORT checklist must be included with all submissions.

Clinical trial registration	This is not a clinical trial study
Study protocol	This is not a clinical trial study
Data collection	Prostate cancer patients were asked before radical prostatectomy to donate part of the prostate gland for future research. They were given proper information about the donation and given the chance to ask questions. This tissue collection and storage was organized by Biobank1, which we subsequently purchased donated tissue from. Clinical follow-up data was collected from patient journals.
Outcomes	This is a retrospective study and none of the outcomes were therefore defined ahead. Nevertheless, we used relapse after radical prostatctomy (confirmed by either increased serum PSA or initiation of prostate cancer treatments) as an outcome measure.
